# Relationship between diagnostic characteristics, lifestyle habits and breast cancer subtypes in women^*^

**DOI:** 10.1590/1518-8345.7492.4581

**Published:** 2025-07-11

**Authors:** Fernanda Cristina Marin, Clóvis Arlindo de Sousa, Erika de Sá Vieira Abuchaim, Isília Aparecida Silva

**Affiliations:** 1 Universidade de São Paulo, Escola de Enfermagem, São Paulo, SP, Brazil.; 2 Universidade Regional de Blumenau, Departamento de Educação Física e Desportos, Blumenau, SC, Brazil.; 3 Universidade Federal de São Paulo, Escola Paulista de Enfermagem, São Paulo, SP, Brazil.

**Keywords:** Breast Neoplasms, Habits, Diet, Alcohol Drinking, Tobacco Use Disorder, Exercise

## Abstract

to investigate the relationship between diagnostic characteristics, lifestyle, habits and the occurrence of breast cancer subtypes in adult and elderly women.

cross-sectional study of women over 18 years old undergoing treatment or follow-up for breast cancer at a cancer institute in the state of São Paulo. Data were collected between December 2022 and July 2023 through telephone interviews and a survey of electronic medical records. Statistical analysis included absolute and relative frequency distribution and bivariate analysis, using the Chi-square test or Fisher’s Exact Test, considering statistically significant values with p≤0.05, with a 95% confidence interval.

323 women took part in the study. Women who did not consume processed foods, soft drinks, or artificial juices had a lower proportion of ductal carcinoma. And those aged <40 had a higher proportion of the triple negative breast cancer subtype.

the results indicate a significant relationship between age at diagnosis and molecular subtype, as well as relevant patterns between the consumption of industrialized foods and soft drinks or artificial juices and the histological subtypes of the disease. These findings highlight the importance of considering such factors in the prevention of the disease.

## Highlights

**(1)** Significant relationship between age at diagnosis and molecular subtype. **(2)** Significant relationship between consumption of processed foods and histological subtypes. **(3)** Significant relationship between consumption of soft drinks or juices and histological subtypes. **(4)** To guide public health and breast cancer prevention strategies.

## Introduction

According to the World Health Organization (WHO), in 2020, a total of 2.3 million women were diagnosed with breast cancer, and, in the same period, 685,000 died worldwide from breast cancer^(1)^. The Global Cancer Observatory (Globocan) corroborates the seriousness of this scenario by estimating that 23.8% of the 20 million new cases of cancer registered worldwide were breast cancer among women^(2)^.

In Brazil, the National Cancer Institute (INCA) estimates that in the three-year period 2023-2025 there will be 704,000 new cases of cancer per year, with breast cancer in women accounting for 73,610 of these^(3)^. These figures highlight the need for effective actions to control and prevent this neoplasm in the female population.

From a histological point of view, breast cancer can be classified as ductal carcinoma (originating in the mammary ducts) or lobular carcinoma (located in the milk-producing lobules)^(4)^. In addition to these, the American Cancer Society (ACS) recognizes four other special subtypes of breast neoplasm: inflammatory carcinoma (characterized by inflammatory signs on the skin), mammary angiosarcoma (a rare subtype that begins in the lining cells of blood or lymphatic vessels), phyllodes tumor (developed in the connective tissue of the breast) and Paget’s disease (begins in the mammary ducts and spreads to the skin of the nipple and areola)^(4)^.

The molecular subtypes of breast cancer, categorized on the basis of distinct genetic characteristics, include: luminal (hormone receptors for estrogen and progesterone positive), HER-2 enriched (human epidermal growth factor receptor 2 - HER2) and basal (negative for estrogen, progesterone and HER2 receptors), also known as triple negatives (TNs)^(4)^. The luminal subtypes were defined by the 12th St. Gallen Conference as luminal A, characterized by being positive for estrogen receptor (ER) and/or progesterone receptor (PR), negative for HER2, and with a low cell proliferation index (Ki-67<14%)^(5)^. Luminal subtype B includes cases that are positive for ER and/or PR and negative for HER2, but with a high Ki-67 index, or that are positive for ER and/or PR and HER2, regardless of the Ki-67 index^(5)^.

According to the ACS, the luminal A subtype is responsible for approximately 68% of all breast cancer cases^(6)^. The luminal B and triple negative subtypes each account for approximately 10% of cases^(6)^. It is important to note that the triple negative subtype has an incidence of approximately 20% among African-American women and is more prevalent in young women and those with BRCA1 gene mutations. Finally, the HER2-enriched subtype is the least common, accounting for approximately 4% of all breast cancer diagnoses^(6)^.

Considering this distribution of breast cancer subtypes, a study of 137 young women (mean age 34.7 years) diagnosed with breast cancer revealed that 11.4%^(15)^ were stage four at the time of diagnosis. Among these patients, invasive ductal carcinoma was the predominant histological type, identified in 86% (118) of the cases. The distribution of molecular subtypes identified was 16.8% for luminal A, 38.7% for luminal B, 30.5% for HER2-enriched, and 15.3% for triple-negative, reflecting the variation in the incidence of subtypes in a young population^(7)^.

With regard to the main risk factors associated with breast cancer, the National Cancer Institute (NIH) points out that environmental factors, including lifestyle habits, can influence both its development and prevention^(8)^. According to the NIH, the relationship between dietary factors and cancer is complex and often inconclusive, citing as an example the fact that diets rich in fruit, vegetables, and calcium are associated with a lower risk of cancer, although there is no definitive consensus in this regard^(8)^. A systematic review study found a weak association between the consumption of unprocessed red meat and a 6% increase in the risk of colorectal cancer and a 3% increase in breast cancer^(9)^.

The NIH also points out that alcohol consumption is associated with an increased risk of several types of cancer, including breast, liver, and colorectal cancer in women, and that smoking is a possible risk factor for breast cancer, slightly increasing the risk of diagnosis, especially in young smokers with high consumption, with exposure to passive smoking being an additional risk factor, particularly in childhood and for premenopausal breast cancer^(8)^.

On the other hand, regular physical activity is associated with a 10% to 20% reduction in the risk of breast cancer, being inversely proportional and independent of body mass index (BMI)^(8)^. However, obesity is recognized as a risk factor for several types of cancer, including postmenopausal breast cancer^(10)^. A study published in 2021 suggests a strong positive association between high BMI and ER/PR+HER2- and HER2+ breast cancer subtypes in postmenopausal women compared to premenopausal women^(11)^. According to the ACS, the risk of postmenopausal hormone receptor-positive breast cancer is 1.5 to 2 times higher in overweight or obese women, possibly due to higher levels of estrogen from adipose tissue and other factors such as high insulin levels^(6)^.

The possible associations between lifestyle habits and the different subtypes of breast cancer are still poorly investigated and, in this context, this study aims to analyze the prevalence and verify the relationships between lifestyle habits, diagnostic characteristics, and the occurrence of different subtypes of breast cancer in adult and elderly women.

## Method

### Type of study

This is a cross-sectional study.

### Location

The study was carried out at São Paulo State Cancer Institute (ICESP), located in the central region of São Paulo and part of the *Hospital das Clínicas* complex of the University of São Paulo Medical School (HCFMUSP).

### Period

Data collection took place from December 2020 to July 2023.

### Population

Women aged 18 or older diagnosed with any subtype of breast cancer and undergoing treatment or follow-up at the institution took part in the study. There were no restrictions on the time since diagnosis or the maximum age of the participants. Exclusion criteria included patients who, at the time of data collection, had physical, cognitive or emotional limitations that prevented them from taking part in the study.

The sample selection was based on a report from ICESP’s Health Information Management Sector (GIS), which provided the name and telephone contact of 1,881 women treated in the chemotherapy, radiotherapy, rehabilitation, and outpatient consultation sectors between June 2019 and May 2020.

The sample calculation was carried out based on the sample size for frequency in a population, population size (for the finite population correction factor) (N): 1881; Hypothetical % frequency of the outcome factor in the population (p): 50%+/-5%; Confidence limits as % of 100 (absolute+/-%) (d): 5%; and Design effect (for group surveys-EDFF):1. The calculated sample size was 320 women, with a 95% confidence interval.

### Data collection instrument

The collection instrument was developed specifically for this study and is structured into two main parts: the first covers sociodemographic characteristics and lifestyle habits, collected through individual interviews conducted by telephone, and the second diagnostic characteristics, obtained by consulting electronic medical records. To guarantee the content validity and accuracy of the instrument, the research team, made up of breastfeeding and breast cancer specialists, worked together to construct and revise the questions based on the results of the pilot test. This collaborative process was crucial to the construction and revision of the questions, ensuring their relevance and alignment with the research objectives. Initially drafted in Microsoft Word, the instrument was transferred to RedCap software.

After approval by the Research Ethics Committees (RECs), a pilot test was carried out to assess the consistency, applicability and effectiveness of the data collection instrument. The process made it possible to check the clarity of the questions and women’s understanding, adjust the format of the interviews and estimate the average time needed for collection. It also enabled the flow of communication to adequately cover the topic in order to achieve the study’s objectives. During this stage, eight interviews were carried out, the results of which indicated that the instrument and the communication dynamics were suitable and no changes were necessary. The interviews were incorporated into the research, demonstrating the usefulness and robustness of this initial stage.

The pilot test was fundamental in confirming the consistent and careful design of the instrument, demonstrating the reliability and validity of the data collection process. This initial procedure not only validated the instrument, but ensured that the questionnaire met the objectives of the study.

### Variables

The sample was characterized using the following variables and categorizations: age at the time of the interview (<40 years, 40 to 59 years and 60+ years), self-reported race (yellow, white, brown, black, didn’t know/didn’t want to say), schooling (illiterate, elementary school, secondary school, higher education and postgraduate), marital status (single, married, stable union, widowed, separated or divorced, didn’t want to say), region where they were born (North, Northeast, Midwest, South, Southeast), region where they have lived most of their lives (North, Northeast, Midwest, South, Southeast) and family income (categorized in minimum wage in force at the time - R$ 1,039.00 - less than two, two to four, five or more).

The lifestyle habits considered as independent variables in this study were: alcohol consumption (yes or no), tobacco consumption (never, ex-smoker or smoker), physical activity (insufficiently active - <150 min/week or sufficiently active - >= 150 min/week) and the type of diet that predominates in most of life.

Also considered as an independent variable, the predominant dietary pattern was assessed by investigating the daily consumption of different types of food: industrialized products (sausages, canned and/or processed products), sugar, milk (and dairy products), vegetables (and fruit) and soft drinks or artificial juices. All were categorized individually as “Yes” or “No”. If yes, the frequency of intake was detailed, classifying it as daily, weekly, monthly or rare.

Also as an independent variable, the diagnostic characteristics: age at diagnosis (<40 years, 40 to 59 years and 60+ years), body mass index (BMI) at diagnosis (normal <25.0, overweight 25.0 to 29.9 and obese>=30 kg/m²) and family history of cancer (yes or no). If yes, the specific type of cancer and the degree of kinship of the affected individuals were detailed.

As the study’s dependent variable, breast cancer was classified into histological subtypes (ductal carcinoma, lobular carcinoma or others) or molecular subtypes (luminal A, luminal B, HER2+ or TN).

The outcome was the occurrence of the different subtypes of breast cancer in relation to the participants’ lifestyle habits and diagnostic characteristics.

### Data collection

The list containing the names and telephone numbers of the 1,881 women given in the ICESP report was given only once and used throughout the data collection procedure. Every week, following the alphabetical order of the names on this list, 15 women were selected to be invited to take part in the study. It is important to note that this sample (15 women/week) was adjusted according to the number of responses obtained, and modified as necessary.

Data collection took place at two different times: 1. Presentation of the study and interview and 2. Consultation of the patient’s institutional electronic medical record. During the telephone contact, the study was presented in detail and the Informed Consent Form was read out and recorded. Women who agreed to take part in the study were interviewed by the main researcher or a collaborator (a nurse specialized in breastfeeding), trained for this purpose. All the interviews were recorded and stored securely on the principal investigator’s computer, with a password that only she knew. Access to the electronic medical records, in order to gather the data to be used in the study, was carried out exclusively by the researcher within the institution.

### Data analysis

The RedCap platform database was used to record the data, which was organized, statistically processed and analyzed using the Statistical Package for the Social Sciences - SPSS, version 22.

Descriptive statistics were carried out, with absolute and relative frequency distribution for all variables and presented in tables and graphs. For bivariate analysis with breast cancer, the Chi-Square test or Fisher’s Exact Test was used when more than 20% of the expected frequencies were less than five. For all statistical analyses, a significance level of p≤0.05 was adopted, corresponding to a 95% confidence interval. The prevalence ratio was used as a measure of effect, providing a quantification of the associations between lifestyle habits and the incidence of the various subtypes of breast cancer. Adjusted standardized residuals with values greater than +2.0 or less than - 2.0 were considered to identify statistical differences between the categories of the associated variable.

### Ethical aspects

This research followed the ethical precepts for research involving human beings, established by Resolution 466/12 of the National Health Council^(12)^.

In accordance with current requirements, this project was submitted to the Research Ethics Committee of the USP School of Nursing (EEUSP) - opinion number 4.341.047. It underwent the evaluation and authorization procedures of the CEP at the *Hospital das Clínicas* of the University of São Paulo (CEP-HCFMUSP) - opinion number 4.303.589 - in accordance with its institutional requirements.

## Results

**Figure 1 -:**
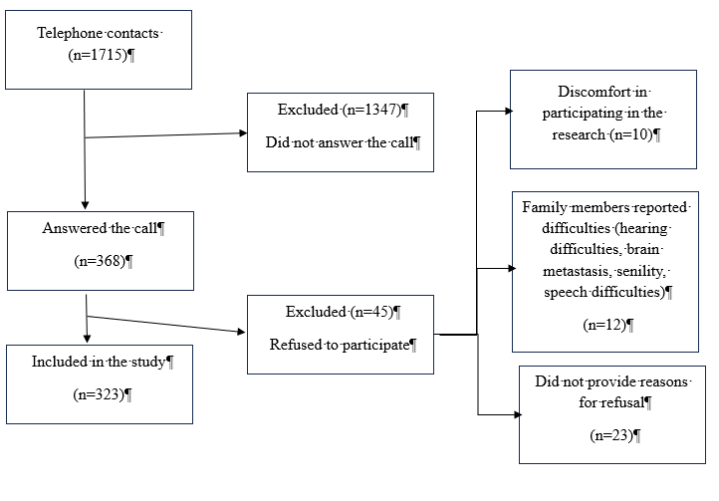
Flowchart of telephone contacts

Telephone contacts were made according to the flowchart in Figure 1.

The study included 323 women aged between 26 and 86. The majority, 213 (65.9%), were aged between 40 and 59 and self-declared as white (174, 53.9%). The predominant level of education was secondary or higher education (110, 34.1%, and 114, 35.3%, respectively). Of the women interviewed, 139 (43%) were married, the majority (237, 73.4%) were from the Southeast and 301 (93.2%) had spent most of their lives in this region.

The family income of 69 (21.4%) of the women was less than two minimum wages, while 150 (46.4%) earned up to four minimum wages. As for lifestyle habits, the majority, 297 (92%) did not consume alcohol and 198 (61.3%) had never smoked. Only 42 (13%) practiced physical activity regularly. As for food preferences throughout their lives, 271 (83.9%) of the participants reported consuming processed foods every day. In addition, 250 (77.4%) reported eating sugar on a daily basis, either to sweetened drinks such as coffee and juices, or as an integral part of sweet foods. In addition, 196 (60.9%) of the women reported daily consumption of soft drinks or artificial juices.

At the time of diagnosis, the majority (206, 63.8%) were between 40 and 59 years old and 126 (39%) had a body mass index between 25 and 29.9. In addition, 124 (38.4%) reported a family history of breast cancer. Ductal carcinoma was the most common histological type (283, 87.6%), while 28 (8.7%) were diagnosed with lobular carcinoma and other less frequent subtypes (mucinous carcinoma, metaplastic carcinoma, medullary carcinoma and micropapillary carcinoma). The luminal B molecular subtype was predominant (140, 44.2%), while triple negative was the least common (50, 15.8%).

The analysis of diagnostic characteristics in relation to histological subtypes, as shown in Table 1, did not reach statistical significance.

**Table 1 -:** Relationship between breast cancer-related characteristics of adult and elderly women and histological subtypes of breast cancer. São Paulo, SP, Brazil, 2023

**Age at diagnosis (years)**	**Histological subtype**				**Total n=323**	**p-value**
		**Ductal carcinoma**	**Lobular carcinoma**	**Others**		
<40	n	73	3	2	78	0.253*
	%	93.6	3.8	2.6	100.0	
	Residual	1.8	-1.7	-0.6		
40 - 59	n	178	19	9	206	0.253*
	%	86.4	9.2	4.4	100.0	
	Residual	-0.9	0.5	0.8		
60+	n	32	6	1	39	0.253*
	%	82.1	15.4	2.6	100.0	
	Residual	-1.1	1.6	-0.4		
**BMI** ^†^					n=323	
<25.0	n	95	10	5	110	0.652*
	%	86.4	9.1	4.5	100.0	
	Residual	-0.5	0.2	0.6		
25.0 - 29.9	n	113	8	5	126	0.652*
	%	89.7	6.3	4.0	100.0	
	Residual	0.9	-1.2	0.2		
>=30	n	75	10	2	87	0.652*
	%	86.2	11.5	2.3	100.0	
	Residual	-0.5	1.1	-0.8		
**Total family members with breast cancer**					n=323	
0	n	172	18	9	199	0.609*
	%	86.4	9.0	4.5	100.0	
	Residual	-0.8	0.3	1		
1	n	73	5	3	81	0.609*
	%	90.1	6.2	3.7	100.0	
	Residual	0.8	-0.9	0		
2+	n	38	5	0	43	0.609*
	%	88.4	11.6	0.0	100.0	
	Residual	0.2	0.7	-1.4		

*Fisher’s Exact Test; ^†^BMI = Body mass index

As shown in Table 2, the analysis of the possible relationships between the participants’ lifestyle habits and the histological subtypes of breast cancer showed a statistically significant difference between the daily consumption of processed foods and the histological subtypes of breast cancer (p=0.003). Of the participants who consumed processed foods on a daily basis, the majority (243, 89.7%) had ductal carcinoma. The consumption of soft drinks or artificial juices was also statistically significant (p=0.044) in relation to the histological subtypes of breast cancer

**Table 2 -:** Relationship between lifestyle habits and histological subtypes of breast cancer in adult and elderly women. São Paulo, SP, Brazil, 2023

		**Histological subtype**			**Total**	**p-value**
		**Ductal carcinoma**	**Lobular carcinoma**	**Others**		
**Alcohol consumption**					n=323	
No	n	261	26	10	297	0.454*
	%	87.9	8.8	3.4	100.0	
	Residual	0.5	0.2	-1.1		
Yes	n	22	2	2	26	0.454*
	%	84.6	7.7	7.7	100.0	
	Residual	-0.5	-0.2	1.1		
**Smoking**					n=323	
Never	n	172	16	10	198	0.554*
	%	86.9	8.1	5.1	100.0	
	Residual	-0.5	-0.5	1.6		
Ex-smoker	n	75	7	1	83	0.554*
	%	90.4	8.4	1.2	100.0	
	Residual	0.9	-0.1	-1.4		
Smoker	n	36	5	1	42	0.554*
	%	85.7	11.9	2.4	100.0	
	Residual	-0.4	0.8	-0.5		
**Smoking time (years)**					n=125	
1 - 9	n	28	3	1	32	0.648*
	%	87.5	9.4	3.1	100.0	
	Residual	-0.3	-0.1	0.8		
10 - 19	n	26	1	0	27	0.648*
	%	96.3	3.7	0.0	100.0	
	Residual	1.4	-1.2	-0.7		
20 or more	n	57	8	1	66	0.648*
	%	86.4	12.1	1.5	100.0	
	Residual	-0.9	1	-0.1		
**Physical activity (minutes per week)**					n=323	
Insufficiently active (<150)	n	104	11	5	281	1.000*
	%	86.7	9.2	4.2	100.0	
	Residual	-0.6	0.4	0.5		
Sufficiently active (>=150)	n	38	3	1	42	1.000*
	%	90.5	7.1	2.4	100.0	
	Residual	0.6	-0.4	-0.5		
**Predominant type of diet throughout life**						
**Processed foods (daily intake)**					n=323	
No	n	40	6	6	52	0.003
	%	76.9	11.5	11.5	100.0	
	Residual	-2.6	0.8	3.3		
Yes	n	243	22	6	271	0.003
	%	89.7	8.1	2.2	100.0	
	Residual	2.6	-0.8	-3.3		
**Sugar (daily intake)**					n=323	
No	n	62	8	3	73	0.694*
	%	84.9	11.0	4.1	100.0	
	Residual	-0.8	0.8	0.2		
Yes	n	221	20	9	250	0.694*
	%	88.4	8.0	3.6	100.0	
	Residual	0.8	-0.8	-0.2		
**Dairy products (daily intake)**					n=323	
No	n	67	7	3	77	0.952*
	%	87.0	9.1	3.9	100.0	
	Residual	-0.2	0.2	0.1		
Yes	n	216	21	9	246	0.952*
	%	87.8	8.5	3.7	100.0	
	Residual	0.2	-0.2	-0.1		
**Vegetables and fruit (daily intake)**					n=323	
No	n	30	3	0	33	0.752*
	%	90.9	9.1	0.0	100.0	
	Residual	0.6	0.1	-1.2		
Yes	n	253	25	12	290	0.752*
	%	87.2	8.6	4.1	100.0	
	Residual	-0.6	-0.1	1.2		
**Soft drinks or artificial juice (weekly frequency)**					n=322*	
0	n	103	15	8	126	0.044
	%	81.7	11.9	6.3	100.0	
	Residual	-2.5	1.6	2		
1 - 2	n	89	8	0	97	0.044
	%	91.8	8.2	0.0	100.0	
	Residual	1.5	-0.2	-2.3		
3+	n	90	5	4	99	0.044
	%	90.9	5.1	4.0	100.0	
	Residual	1.2	-1.5	0.2		

*Fisher’s Exact Test

The analysis of the relationship between characteristics related to breast cancer and the molecular subtypes of tumors presented by the women, shown in Table 3, showed a statistically significant difference between age at diagnosis and the molecular subtypes of breast cancer (p=0.006). Women under the age of 40 predominantly presented luminal B and TN subtypes, while those aged between 40 and 59 showed a higher prevalence of luminal B and luminal A subtypes.

**Table 3 -:** Relationship between characteristics related to breast cancer and the molecular subtypes of breast cancer presented by the women participating in the study. São Paulo, SP, Brazil, 2023

		Molecular subtype*				**Total**	**p-value**
		**Luminal A**	**Luminal B**	**HER 2+**	**Triple negative**		
**Age at diagnosis (years)**						n=317	
<40	n	13	29	16	20	78	0.006
	%	16.7	37.2	20.5	25.6	100.0	
	Residual	-1.7	-1.3	0.9	2.8		
40 - 59	n	46	94	34	28	202	0.006
	%	22.8	46.5	16.8	13.9	100.0	
	Residual	-0.5	1.4	-0.1	-1.2		
60+	n	16	15	4	2	37	0.006
	%	43.2	40.5	10.8	5.4	100.0	
	Residual	3	-0.4	-1.1	-1.8		
**BMI** ^†^						n=317	
<25.0	n	27	48	14	17	106	0.389 ^‡^
	%	25.5	45.3	13.2	16.0	100.0	
	Residual	0.5	0.4	-1.3	0.1		
25.0 - 29,9	n	32	49	27	16	124	0.389 ^‡^
	%	25.8	39.5	21.8	12.9	100.0	
	Residual	0.7	-1.2	1.8	-1.1		
>=30	n	16	41	13	17	87	0.389 ^‡^
	%	18.4	47.1	14.9	19.5	100.0	
	Residual	-1.4	0.8	-0.6	1.1		
**Family members with breast cancer**						n=317	
0	n	41	84	35	36	196	0.048 ^‡^
	%	20.9	42.9	17.9	18.4	100.0	
	Residual	-1.5	-0.3	0.5	1.6		
1	n	22	39	13	4	78	0.048 ^‡^
	%	28.2	50.0	16.7	5.1	100.0	
	Residual	1.1	1.3	-0.1	-3		
2+	n	12	15	6	10	43	0.048 ^‡^
	%	27.9	34.9	14.0	23.3	100.0	
	Residual	0.7	-1.2	-0.6	1.4		

*Records without immunohistochemistry data for 6 women; ^†^BMI = Body mass index; ^‡^Fisher’s exact test

As shown in Table 4, no statistically significant associations were found between the molecular subtypes of breast cancer and the lifestyle habits of the women investigated.

**Table 4 -:** Relationship between lifestyle habits and the molecular subtypes of breast cancer presented by the women participating in the study. São Paulo, SP, Brazil, 2023

		Molecular subtype*				Total	p-value
		Luminal A	Luminal B	HER 2+	Triple negative	n=317	
**Alcohol consumption**							
No	n	66	127	53	46	292	0.189 ^†^
	%	22.6	43.5	18.2	15.8	100.0	
	Residual	-1.5	0	1.8	0		
Yes	n	9	11	1	4	25	0.189 ^†^
	%	36.0	44.0	4.0	16.0	100.0	
	Residual	1.5	0	-1.8	0		
**Smoking**						n=317	
Never	n	42	85	33	35	195	0.695 ^†^
	%	21.5	43.6	16.9	17.9	100.0	
	Residual	-1.1	0	-0.1	1.3		
Ex-smoker	n	22	37	14	8	81	0.695 ^†^
	%	27.2	45.7	17.3	9.9	100.0	
	Residual	0.9	0.5	0.1	-1.7		
Smoker	n	11	16	7	7	41	0.695 ^†^
	%	26.8	39	17.1	17.1	100.0	
	Residual	0.5	-0.6	0	0.2		
**Smoking time (years)**						n=122	
1 - 9	n	6	20	3	3	32	0.290 ^†^
	%	18.8	62.5	9.4	9.4	100.0	
	Residual	-1.2	2.5	-1.4	-0.6		
10 - 19	n	7	11	4	4	26	0.290 ^†^
	%	26.9	42.3	15.4	15.4	100.0	
	Residual	0	-0.1	-0.3	0.5		
20 or more	n	20	22	14	8	64	0.290 ^†^
	%	31.3	34.4	21.9	12.5	100.0	
	Residual	1.1	-2.1	1.4	0.1		
**Physical activity (minutes per week)**						n=317	
Insufficiently active (<150)	n	65	118	48	46	277	0.646 ^†^
	%	28.8	40.7	13.6	16.9	100.0	
	Residual	0.5	-1	-0.2	1.1		
Sufficiently active (>=150)	n	10	20	6	4	40	0.646 ^†^
	%	25	50	15	10	100.0	
	Residual	-0.5	1	0.2	-1.1		
**Predominant type of diet throughout life**							
**Processed foods (daily intake)**						n=317	
No	n	13	23	8	8	52	0.992 ^†^
	%	25	44.2	15.4	15.4	100.0	
	Residual	0.2	0.1	-0.3	-0.1		
Yes	n	62	115	46	42	265	0.992 ^†^
	%	23.4	43.4	17.4	15.8	100.0	
	Residual	-0.2	-0.1	0.3	0.1		
**Sugar (daily intake)**						n=317	
No	n	22	33	6	9	70	0.072 ^†^
	%	31.4	47.1	8.6	12.9	100.0	
	Residual	1.7	0.7	-2.1	-0.8		
Yes	n	53	105	48	41	247	0.072 ^†^
	%	21.5	42.5	19.4	16.6	100.0	
	Residual	-1.7	-0.7	2.1	0.8		
**Dairy products (daily intake)**						n=317	
No	n	18	35	10	14	77	0.690 ^†^
	%	23.4	45.5	13.0	18.2	100.0	
	Residual	-0.1	0.4	-1.1	0.7	-0.1	
Yes	n	57	103	44	36	240	0.690 ^†^
	%	23.8	42.9	18.3	15.0	100.0	
	Residual	0.1	-0.4	1.1	-0.7		
**Vegetables and fruit (daily intake)**						n=317	
No	n	6	13	7	7	33	0.607 ^†^
	%	18.2	39.4	21.2	21.2	100.0	
	Residual	-0.8	-0.5	0.7	0.9		
Yes	n	69	125	47	43	284	0.607 ^†^
	%	24.3	44.0	16.5	15.1	100.0	
	Residual	0.8	0.5	-0.7	-0.9		
**Soft drinks or artificial juice (weekly frequency)**						n=316 ^‡^	
0	n	36	53	16	19	124	0.451 ^†^
	%	29.0	42.7	12.9	15.3	100.0	
** **	Residual	1.8	-0.3	-1.5	-0.2	93	
1 a 2	n	17	40	21	15	100.0	0.451 ^†^
	%	18.3	43.0	22.6	16.1		
	Residual	-1.5	-0.2	1.8	0.1	99	
3+	n	22	45	16	16	100.0	0.451 ^†^
	%	22.2	45.5	16.2	16.2		
	Residual	-0.4	0.4	-0.2	0.1		

*Record without immunohistochemistry data for 6 women; ^†^Fisher’s exact test; ^‡^No response from 1 woman

## Discussion

Analysis of the data obtained in this study involving 323 women revealed significant associations between demographic characteristics, dietary habits, and breast cancer subtypes. We observed that not consuming industrialized foods, as well as not consuming soft drinks or artificial juices was associated with a lower proportion of ductal carcinoma. In addition, women under the age of 40 had a higher occurrence of the triple-negative breast cancer subtype. These findings reinforce the influence of age on the molecular profile of breast cancer and highlight the relevance of dietary factors in the different histological subtypes, underlining the need to integrate these variables when developing prevention strategies and effective health policies for managing the disease.

However, when interpreting these results, it is essential to consider the limitations of the study. The collection of information, based on self-reports, presents vulnerabilities such as measurement inaccuracies, and possible memory biases, which may vary according to the age group of the interviewees. In addition, the representativeness of the sample should be viewed with caution, as it may not fully reflect the general population, making it difficult to generalize the results. Survival bias is another relevant concern, since the exclusive inclusion of diagnosed and treated women can distort the relationship observed between lifestyle habits, diagnostic characteristics and the occurrence of breast cancer subtypes. This can lead to overestimation of associations, as cases with unfavorable outcomes may be under-represented.

Data collection was also affected by external factors, such as the pandemic caused by the SARS-CoV virus. The need to adapt the collection procedures by submitting changes to the research ethics committees (REC) of the School of Nursing and the USP Medical School Foundation led to delays and eliminated the possibility of conducting face-to-face interviews. This modification limited direct contact between the researcher and participants, potentially influencing the depth and quality of the information collected.

Despite these limitations, the findings on the distribution of breast cancer types are consistent with population data. The majority (87.6%) of cases were ductal carcinoma, a result in line with the general prevalence of this type in the population^(13)^. Other histological subtypes, such as mucinous carcinoma, metaplastic carcinoma, medullary carcinoma, and micropapillary carcinoma, were also identified, although they were rare, each accounting for less than 5% of cases, as described in the literature^(4)^.

With regard to molecular subtypes, the predominance of luminal tumors in our study reinforces the global evidence that they account for around 80% of breast cancer cases, while the triple-negative (TN) subtype comprises approximately 10-15% of diagnoses^(14)^. This agreement with international data strengthens the validity of our results, indicating that the molecular profile observed corroborates the trend already documented in the literature.

Furthermore, analysis of the “age at diagnosis” variable revealed that most of the study participants were aged between 40 and 59. According to INCA, the highest concentration of screening tests, such as mammography, occurs among women aged 50 to 59, followed by the 60 to 69 age group^(3)^. In contrast, the ACS recommends starting screening from the age of 40^(4)^. These differences in recommendations underline the importance of an ongoing discussion about the best age to start screening, taking into account the epidemiological context and the specificities of the population studied.

Our study identified a significant association between age and the molecular subtypes of breast cancer, with an increased risk of triple-negative (TN) cancer in women under 40. This observation is corroborated by a prospective registry in Brazil, which indicated a higher proportion of Luminal B and TN tumors in younger patients^(15)^. In addition, the literature already establishes that TN cancers are diagnosed disproportionately in younger women^(16)^.

These findings highlight the importance of incorporating the diversity of molecular subtypes and the variability in risk between different age groups when drawing up screening guidelines. It is therefore essential to carry out further research into the most appropriate age to start screening, ensuring early and effective detection in different population groups.

In addition, 39% of the women in this study had a body mass index (BMI) between 25 and 29.9 kg/m², while the 2023 report from the Food and Nutrition Surveillance System identified that 33.7% of the Brazilian adult female population is overweight^(17)^. Despite this, the analysis did not reveal a statistically significant association between BMI and the histological or molecular subtypes of breast cancer. These results are in line with another study which, although recognizing the relationship between high BMI and the general risk of breast cancer, found no significance when examining this relationship in relation to specific subtypes of the disease^(18)^. It should be noted that the literature still lacks more detailed studies on these associations.

With regard to factors such as alcohol consumption, smoking and physical activity, we did not identify statistically significant associations in our study, differing from what is widely documented. For example, an analysis of an American prospective cohort showed a significant correlation between alcohol consumption and an increased risk of hormone receptor-positive tumors, especially lobular carcinoma, with more pronounced effects in postmenopausal women^(19)^. These findings are in line with the idea that hormonal mechanisms influence the relationship between alcohol and breast cancer, highlighting the modulatory role of estrogen^(19)^.

As for smoking, a comprehensive meta-analysis involving data from 14 cohort studies revealed a modest overall increase (7%) in the risk of breast cancer among women who smoked at the time of the study^(20)^. This association was particularly relevant in cancers expressing the estrogen receptor (ER), with an increased risk of 76% for ER-positive tumors^(20)^. Furthermore, the impact of smoking seems to vary according to the age of onset. Women who started smoking before their first pregnancy had an even higher risk of developing breast cancer^(20)^.

These results highlight the complexity of the interactions between lifestyle factors and breast cancer risk, suggesting that age and timing of exposure to factors such as tobacco play key roles in breast carcinogenesis. Therefore, future research is needed to better understand these dynamics and inform personalized preventive strategies.

Contrary to the findings of the present study, which found no statistical significance in the relationship between physical activity and the histological or molecular subtypes of breast cancer, a case-control study carried out in Germany showed a significant inverse association between physical activity and the risk of postmenopausal breast cancer^(21)^. The authors reported that the protective effect was more pronounced in hormone receptor-positive invasive carcinomas^(21)^. In addition, it was found that physical activity performed after the age of 50 was more effective in preventing RE+ and RP+ carcinomas in postmenopausal women than at a younger age^(21)^.

In this study, we were able to establish statistical significance in the relationship between histological subtypes and some variables related to dietary habits, specifically the intake of processed foods (p=0.003) and the consumption of soft drinks or artificial juices (p=0.044) (Table 2). With regard to diet, the scientific literature presents heterogeneous results. Some studies suggest that the aromatase enzyme, present in adipose tissue, can convert circulating cholesterol into estradiol, increasing the levels of this hormone in breast tissue and influencing the expression of estrogen receptors, which can affect the behavior of cancer cells^(22)^. In addition, studies show that diets rich in cured foods may be associated with an increased risk of hormone receptor-negative breast cancer, while consumption of grilled foods correlates with an increased risk of hormone receptor-positive breast cancer^(23)^.

The role of industrialized foods also deserves to be highlighted. These foods, including processed and sausage products, contribute to a pro-inflammatory diet, while a diet rich in fruit, vegetables and whole grains is associated with lower levels of inflammatory biomarkers^(24)^. On the other hand, high consumption of red and processed meat is related to an increase in these inflammatory markers^(24)^. In this study, we observed significant differences in the consumption of processed foods associated with ductal carcinoma. Specifically, the frequency of ductal carcinoma was lower among women who reported abstaining from processed foods and from soft drinks or artificial juices, with residual values of -2.6 and -2.5, respectively (p=0.044). This suggests that not consuming these products may be associated with a lower incidence of ductal carcinoma.

However, we found no statistical significance when analyzing the associations between the consumption of processed foods, soft drinks or artificial juices, and the molecular subtypes. Although other studies have reported a relationship between pro-inflammatory diets and breast cancer subtypes positive for the estrogen or progesterone receptor, as well as for the HER2 receptor, this association was not observed in the case of the triple-negative (TN) subtype^(24)^.

It is important to note that the literature is still scarce when it comes to characterizing the histological subtypes of breast cancer, with greater emphasis being placed on the molecular subtypes. Although this study is cross-sectional in nature, which prevents inferring causality, our findings provide significant evidence that contributes to the generation of hypotheses and the identification of risk factors or markers that deserve further investigation. Cross-sectional studies such as this one are fundamental for raising hypotheses and suggesting directions for future research, especially through longitudinal or experimental studies, which could clarify causal relationships.

In summary, even though the observed associations do not establish a direct cause and effect relationship, the results suggest relevant connections between lifestyle habits, diagnostic characteristics and breast cancer subtypes. These findings have the potential to guide future research and contribute significantly to the advancement of knowledge in the area. In addition, our results offer valuable support for nursing practice, highlighting the importance of promoting healthy lifestyles and providing qualified care to women undergoing treatment or follow-up for breast cancer. We emphasize the need for further studies, especially in the Brazilian population, to develop effective preventive strategies against the disease.

## Conclusion

This study sought to investigate the complex relationship between women’s characteristics related to breast cancer, their lifestyle habits and the incidence of different subtypes of the disease. The results obtained make a significant contribution to understanding these interactions and their influence on the manifestation of breast cancer subtypes.

A significant association was found between women’s age at diagnosis and the molecular subtypes of breast cancer determined by immunohistochemistry. Specifically, women under the age of 40 were more likely to develop triple negative (TN) breast cancer.

Our findings highlight associations between lifestyle habits, such as the consumption of industrialized foods and soft drinks or artificial juices, and the histological subtypes of breast cancer. This highlights the importance of considering such factors in the prevention of the disease.

This study has made a substantial contribution to the fields of nursing education, research and care, by giving greater visibility to modifiable lifestyle habits identified as risk factors for breast cancer. It provides information to guide public health strategies for prevention, early diagnosis and the implementation of effective policies to mitigate the impact of breast cancer on society.
