# Supplementary material for: Relationship between diagnostic characteristics, lifestyle habits and breast cancer subtypes in women
Source: Rev Lat Am Enfermagem. 2025 Jul 11;33:e4581. doi: 10.1590/1518-8345.7492.4581 (PMC12259224; doi:10.1590/1518-8345.7492.4581)
Supplement: Translation

# Translation: Relação entre características diagnósticas, hábitos de vida e subtipos de câncer de mama em mulheres^*^

**DOI:** 10.1590/1518-8345.7492.4582

**Authors:** Fernanda Cristina Marin, Clóvis Arlindo de Sousa, Erika de Sá Vieira Abuchaim, Isília Aparecida Silva

**Affiliations:** 1 Universidade de São Paulo, Escola de Enfermagem, São Paulo, SP, Brasil.; 2 Universidade Regional de Blumenau, Departamento de Educação Física e Desportos, Blumenau, SC, Brasil.; 3 Universidade Federal de São Paulo, Escola Paulista de Enfermagem, São Paulo, SP, Brasil.

**Keywords:** Neoplasias da Mama, Hábitos, Dieta, Consumo de Bebidas Alcoólicas, Tabagismo, Atividade Física

## Abstract

investigar a relação entre características diagnósticas, hábitos de vida e a ocorrência de subtipos de câncer de mama em mulheres adultas e idosas.

estudo transversal realizado com mulheres ≥ 18 anos em tratamento ou seguimento de neoplasia mamária num Instituto de Câncer do estado de São Paulo. Os dados foram coletados entre dezembro/2022 e julho/2023 por meio de entrevista telefônica e levantamento de prontuários eletrônicos. A análise estatística incluiu distribuição de frequência absoluta e relativa e análise bivariada, utilizando o teste Qui-Quadrado ou o Teste Exato de Fisher, considerando valores estatisticamente significativos com p≤0,05, com intervalo de confiança de 95%.

participaram do estudo 323 mulheres. As mulheres que não consumiam alimentos industrializados, refrigerantes ou sucos artificiais tiveram uma menor proporção de carcinoma ductal. E aquelas com idade < 40 anos tiveram uma maior proporção de ocorrência do subtipo de câncer de mama triplo negativo.

os resultados indicam uma relação significativa entre a idade, o diagnóstico e o subtipo molecular, além de padrões relevantes entre o consumo de alimentos industrializados e refrigerantes ou sucos artificiais e os subtipos histológicos da doença. Essas descobertas ressaltam a importância de considerar tais fatores na prevenção da doença.

## Destaques

**(1)** Relação significativa entre a idade ao diagnóstico e o subtipo molecular. **(2)** Relação significativa entre o consumo de alimentos industrializados e subtipos histológicos. **(3)** Relação significativa entre o consumo de refrigerantes ou sucos e subtipos histológicos. **(4)** Orientar estratégias de saúde pública e prevenção do câncer de mama.

## Introdução

Segundo a Organização Mundial da Saúde (OMS), em 2020, um total de 2,3 milhões de mulheres foram diagnosticadas com câncer de mama e, no mesmo período, 685.000 morreram em todo o mundo em decorrência de neoplasia mamária^(1)^. O *Global Cancer Observatory* (*Globocan*) corrobora a gravidade desse cenário ao estimar que 23,8% dos 20 milhões de novos casos de câncer registrados mundialmente eram de mama entre mulheres^(2)^.

No Brasil, o Instituto Nacional do Câncer (INCA) estima que, no triênio de 2023-2025, serão registrados 704.000 novos casos de câncer por ano, sendo que o câncer de mama em mulheres é responsável por 73.610 desses novos casos^(3)^. Esses dados ressaltam a necessidade de ações eficazes para o controle e a prevenção dessa neoplasia na população feminina.

Do ponto de vista histológico, o câncer de mama pode ser classificado como carcinoma ductal (originado nos ductos mamários) ou carcinoma lobular (localizado nos lóbulos produtores de leite)^(4)^. Além destes, a *American Cancer Society* (ACS) reconhece outros quatro subtipos especiais de neoplasia mamária: carcinoma inflamatório (caracterizado por sinais inflamatórios na pele), angiossarcoma mamário (subtipo raro que se inicia nas células de revestimento dos vasos sanguíneos ou linfáticos), tumor filódio (desenvolvido no tecido conjuntivo da mama) e doença de Paget (começa nos ductos mamários e se espalha para a pele do mamilo e da aréola)^(4)^.

Os subtipos moleculares do câncer de mama, categorizados a partir de características genéticas distintas, incluem os subtipos: luminais (receptores hormonais para estrógeno e progesterona positivos), HER-2 enriquecido (receptor do fator de crescimento epidérmico humano 2 - HER2) e os basais (negativos para os receptores de estrógeno, progesterona e HER2), também conhecidos como triplo negativos (TNs)^(4)^.

Os subtipos luminais foram definidos pela 12ª Conferência de St. Gallen como luminal A, caracterizado por serem positivos para receptores de estrógeno (RE) e/ou progesterona (RP), negativos para HER2, e com baixo índice de proliferação celular (Ki-67<14%)^(5)^. O subtipo luminal B inclui casos positivos para RE e/ou RP e negativos para HER2, mas com alto índice de Ki-67, ou que são positivos para RE e/ou RP e HER2, independentemente do índice de Ki-67^(5)^.

Segundo a ACS, o subtipo luminal A é responsável por aproximadamente 68% de todos os casos de câncer de mama^(6)^. Já os subtipos luminal B e o triplo negativo correspondem, cada um, a aproximadamente 10% dos casos^(6)^. É importante destacar que o subtipo triplo negativo apresenta uma incidência de aproximadamente 20% entre mulheres afro-americanas, sendo mais prevalente em mulheres jovens e naquelas portadoras de mutações do gene BRCA1. Por fim, o HER2-enriquecido é o subtipo menos comum, respondendo por aproximadamente 4% de todos os diagnósticos de câncer de mama^(6)^.

Considerando essa distribuição dos subtipos de câncer de mama, um estudo realizado com 137 mulheres jovens (média de idade de 34,7 anos) diagnosticadas com neoplasia mamária revelou que 11,4% (15) estavam em estágio quatro no momento do diagnóstico. Entre essas pacientes, o carcinoma ductal invasivo foi o tipo histológico predominante, identificado em 86% (118) dos casos. A distribuição dos subtipos moleculares identificados foi de 16,8% para o luminal A, 38,7% para o luminal B, 30,5% para o HER2-enriquecido e 15,3% para o triplo-negativo, refletindo a variação de incidência dos subtipos em uma população jovem^(7)^.

No que se refere aos principais fatores de risco associados ao câncer de mama, o *National Cancer Institute* (NIH) destaca que fatores ambientais, incluindo hábitos de vida, podem influenciar tanto o desenvolvimento quanto a sua prevenção^(8)^. Segundo o NIH, a relação entre fatores dietéticos e o câncer é complexa e frequentemente inconclusiva, citando como exemplo o fato de as dietas ricas em frutas, vegetais e cálcio estarem associadas a um menor risco de câncer, embora não haja consenso definitivo a esse respeito^(8)^. Estudo de revisão sistemática encontrou uma associação fraca entre o consumo de carne vermelha não processada e um aumento de 6% no risco de câncer colorretal e de 3% no câncer de mama^(9)^.

O NIH destaca, ainda, que o consumo de álcool está associado a um aumento do risco de vários tipos de câncer, incluindo câncer de mama, fígado e colorretal em mulheres, e que o tabagismo é um possível fator de risco para o câncer de mama, aumentando ligeiramente o risco para o diagnóstico, especialmente em fumantes jovens e com alto consumo, sendo a exposição ao fumo passivo um fator de risco adicional, particularmente na infância e para o câncer de mama pré-menopáusico^(8)^.

Por outro lado, a prática regular de atividade física está associada a uma redução de 10% a 20% no risco de câncer de mama, sendo inversamente proporcional e independentemente do índice de massa corporal (IMC)^(8)^. Contudo, a obesidade é reconhecida como fator de risco para diversos tipos de câncer, incluindo o câncer de mama na pós-menopausa^(10)^. Estudo publicado em 2021 sugere uma forte associação positiva entre IMC elevado e os subtipos de câncer de mama ER/PR+HER2− e HER2+ em mulheres na pós-menopausa, em comparação com aquelas na pré-menopausa^(11)^. De acordo com a ACS, o risco de câncer de mama receptor hormonal positivo na pós-menopausa é de 1,5 a 2 vezes maior em mulheres com sobrepeso ou obesidade, possivelmente devido a níveis mais altos de estrogênio provenientes do tecido adiposo e outros fatores, como níveis elevados de insulina^(6)^.

As possíveis associações entre os hábitos de vida e os diferentes subtipos de câncer de mama ainda são pouco investigadas e, nesse contexto, o presente estudo tem como objetivo analisar a prevalência e verificar as relações entre hábitos de vida, características diagnósticas e a ocorrência de diferentes subtipos de câncer de mama em mulheres adultas e idosas.

## Método

### Tipo de estudo

Trata-se de um estudo transversal.

### Local

O estudo foi realizado no Instituto do Câncer do Estado de São Paulo (ICESP), localizado na região central da capital paulista e parte do complexo do Hospital das Clínicas da Faculdade de Medicina da Universidade de São Paulo (HCFMUSP).

### Período

A coleta de dados ocorreu no período de dezembro de 2020 a julho de 2023.

### População

Participaram do estudo mulheres com idade igual ou superior a 18 anos, diagnosticadas com qualquer subtipo de câncer de mama, em tratamento ou acompanhamento na instituição. Não foram estabelecidas restrições quanto ao tempo desde o diagnóstico ou à idade máxima das participantes. Como critério de exclusão, foram consideradas inelegíveis pacientes que, no momento da coleta de dados, apresentavam limitações físicas, cognitivas ou emocionais que impedissem a participação no estudo.

A seleção da amostra foi baseada em um relatório do Setor de Gestão de Informação em Saúde (GIS) do ICESP, que forneceu o nome e contato telefônico de 1.881 mulheres atendidas nos setores de quimioterapia, radioterapia, reabilitação e ambulatório de consultas no período de junho de 2019 a maio de 2020.

O cálculo amostral foi realizado com base no tamanho da amostra para frequência em uma população, no tamanho da população (para o fator de correção da população finita) (*N*): 1881; Frequência % hipotética do fator do resultado na população (*p*): 50%+/-5%; Limites de confiança como % de 100 (absoluto+/-%) (*d*): 5%; e Efeito de desenho (para inquéritos em grupo-EDFF):1. O tamanho da amostra calculado foi de 320 mulheres, com intervalo de confiança de 95%.

### Instrumento de coleta de dados

O instrumento de coleta foi desenvolvido especificamente para este estudo, estruturado em duas partes principais: a primeira abrange características sociodemográficas e hábitos de vida, coletadas por meio de entrevistas individuais realizadas via contato telefônico, e a segunda características diagnósticas, obtidas a partir de consulta a prontuários eletrônicos. Para garantir a validade de conteúdo e a acurácia do instrumento, a equipe da pesquisa, composta por especialistas em amamentação e câncer de mama, trabalhou conjuntamente para a construção e revisão das questões com base nos resultados do teste-piloto. Esse processo colaborativo foi crucial para a construção e revisão das questões, assegurando sua pertinência e alinhamento aos objetivos da pesquisa. Inicialmente elaborado no *Microsoft Word*, o instrumento foi transferido para o *softwareRedCap*.

Após a aprovação pelos Comitês de Ética em Pesquisa (CEPs), foi conduzido um teste-piloto com o objetivo de avaliar a consistência, aplicabilidade e eficácia do instrumento de coleta de dados. O processo permitiu verificar a clareza das questões e o entendimento das mulheres, ajustar o formato das entrevistas e estimar o tempo médio necessário para a coleta. Além disso, possibilitou o fluxo de comunicação abrangendo adequadamente o tema para atingir os objetivos do estudo. Durante essa etapa, foram realizadas oito entrevistas, cujos resultados indicaram a adequação do instrumento e da dinâmica de comunicação, não sendo necessárias modificações. As entrevistas foram incorporadas à pesquisa, demonstrando a utilidade e robustez dessa etapa inicial.

O teste-piloto foi fundamental para confirmar a elaboração consistente e criteriosa do instrumento, demonstrar a confiabilidade e a validade do processo de coleta de dados. Esse procedimento inicial não apenas validou o instrumento, mas garantiu que o questionário atendesse aos objetivos do estudo.

### Variáveis

A caracterização da amostra se deu por meio das seguintes variáveis e categorizações: idade no momento da entrevista (<40 anos, 40 a 59 anos e 60+ anos), raça autorreferida (amarela, branca, parda, preta, não sabe/não quis informar), escolaridade (analfabeta, ensino fundamental, ensino médio, ensino superior e pós-graduação), estado conjugal (solteira, casada, união estável, viúva, separada ou divorciada, não quis informar), região onde nasceu (Norte, Nordeste, Centro-Oeste, Sul, Sudeste), região onde viveu a maior parte da vida (Norte, Nordeste, Centro-Oeste, Sul, Sudeste) e renda familiar (categorizada em salário-mínimo vigente à época – R$ 1.039,00 – menos de dois, dois a quatro, cinco ou mais).

Os hábitos de vida, considerados como variáveis independentes no presente estudo foram: consumo de bebida alcoólica (sim ou não), consumo de tabaco (nunca, ex-fumante ou fumante), atividade física (insuficientemente ativa – <150 min/semana ou suficientemente ativa – > = 150 min/semana) e o tipo de alimentação predominante na maior parte da vida.

Considerando também como variável independente, o padrão alimentar predominante foi avaliado mediante a investigação do consumo diário de diferentes tipos de alimentos: produtos industrializados (embutidos, enlatados e ou processados), açúcar, leites (e derivados), legumes (e frutas) e refrigerante ou suco artificial. Todos foram categorizados individualmente como “Sim” ou “Não”. Em casos afirmativos, foi detalhada a frequência da ingestão, classificando-a como diária, semanal, mensal ou rara.

Ainda como variável independente, as características diagnósticas: idade ao diagnóstico (<40 anos, 40 a 59 anos e 60+ anos), índice de massa corporal (IMC) ao diagnóstico (normal <25,0, sobrepeso 25,0 a 29,9 e obesa>=30 kg/m²) e histórico de câncer na família (sim ou não). Em casos afirmativos, foram detalhados o tipo específico de câncer e o grau de parentesco dos indivíduos afetados.

Como variável dependente do estudo, o câncer de mama foi classificado em subtipos histológicos (carcinoma ductal, carcinoma lobular ou outros) ou moleculares (luminal A, luminal B, HER2+ ou TN).

Considerou-se como desfecho a ocorrência dos diferentes subtipos de câncer de mama em relação aos hábitos de vida e características diagnósticas das participantes.

### Coleta de dados

A relação contendo os nomes e telefones das 1.881 mulheres constante do relatório do ICESP foi entregue apenas uma vez e utilizada durante todo o procedimento de coleta de dados. Semanalmente, seguindo a ordem alfabética dos nomes dessa lista, 15 mulheres eram selecionadas para serem convidadas a participar do estudo, o que ocorria por meio de chamada telefônica. É relevante destacar que a amostra (15 mulheres/semana) foi ajustada de acordo com a quantidade de respostas obtidas, sendo modificado conforme necessário.

A coleta de dados ocorreu em dois momentos distintos: 1. Apresentação do estudo e entrevista, e 2. Consulta ao prontuário eletrônico institucional da paciente. Durante o contato telefônico, ocorreu a apresentação detalhada do estudo, leitura e gravação do Termo de Consentimento Livre e Esclarecido. As mulheres que aceitavam participar do estudo eram entrevistadas pela pesquisadora principal, ou colaboradora (enfermeira especializada em amamentação), treinada para esta finalidade. Todas as entrevistas realizadas foram gravadas e armazenadas no computador da pesquisadora principal de maneira segura, garantida por senha que somente a mesma conhecia. O acesso aos prontuários eletrônicos para levantamento dos dados a serem utilizados no estudo foi realizado exclusivamente pela pesquisadora dentro da instituição.

### Análise dos dados

Utilizou-se o banco de dados da plataforma *RedCap* para registro dos dados, os quais foram organizados, tratados estatisticamente e analisados, utilizando-se os recursos do programa *Statistical Package for the Social Sciences* – SPSS, versão 22.

Foi realizada estatística descritiva, com distribuição de frequência absoluta e relativa para todas as variáveis e apresentadas por meio de tabelas e gráficos. Para análise bivariada com câncer de mama, utilizou-se o teste Qui-Quadrado ou o Teste Exato de Fisher, quando mais de 20% das frequências esperadas foram menores do que cinco. Para todas as análises estatísticas, adotou-se um nível de significância de p≤0,05, correspondente a um intervalo de confiança de 95%. A razão de prevalência foi utilizada como medida de efeito, proporcionando uma quantificação das associações entre os hábitos de vida e a incidência dos diversos subtipos de câncer de mama. Resíduos padronizados ajustados com valores superiores a +2,0 ou inferiores a – 2,0 foram considerados para identificar diferença estatística entre as categorias da variável associada.

### Aspectos éticos

Esta pesquisa seguiu os preceitos éticos de pesquisas envolvendo seres humanos, estabelecidos pela Resolução 466/12, do Conselho Nacional de Saúde^(12)^.

De acordo com os requisitos vigentes, este projeto foi submetido ao Comitê de Ética em Pesquisa da Escola de Enfermagem da USP (EEUSP) - número do parecer 4.341.047. Passou pelos trâmites de avaliação e autorização do CEP no Hospital das Clínicas da Universidade de São Paulo (CEP-HCFMUSP) sob número do parecer 4.303.589 – conforme suas exigências institucionais.

## Resultados

Os contatos telefônicos ocorreram conforme o fluxograma da Figura 1.

**Figura 1 -:** Fluxograma dos contatos telefônicos

Assim, o estudo incluiu 323 mulheres com idades entre 26 e 86 anos. A maioria, 213 (65,9%), tinha entre 40 e 59 anos e se autodeclarou branca (174; 53,9%). A escolaridade predominante foi ensino médio ou superior (110; 34,1% e 114; 35,3%, respectivamente). Das mulheres entrevistadas, 139 (43%) eram casadas, a maioria (237, o que corresponde a 73,4%) originária da região Sudeste, sendo que 301 (93,2%) passaram a maior parte de suas vidas nessa região.

A renda familiar de 69 (21,4%) das mulheres era inferior a dois salários-mínimos, enquanto 150 (46,4%) recebiam até quatro salários-mínimos. Quanto aos hábitos de vida, a maioria, 297 (92%) não consumia álcool e 198 (61,3%) nunca fumaram. Apenas 42 (13%) praticavam atividade física regularmente. Quanto à preferência alimentar ao longo da vida, 271 (83,9%) das participantes relataram o consumo diário de alimentos industrializados. Além disso, 250 (77,4%) referiram ingerir açúcar diariamente, seja para adoçar bebidas como café e sucos, ou como parte integrante de alimentos doces. Adicionalmente, 196 (60,9%) das mulheres relataram o consumo diário de refrigerantes ou sucos artificiais.

No momento do diagnóstico, a maioria (206, isto é, 63,8%) tinha entre 40 e 59 anos e 126 (39%) apresentavam índice de massa corporal entre 25 e 29,9. Além disso, 124 (38,4%) relataram histórico familiar de câncer de mama. O carcinoma ductal foi o tipo histológico mais comum (283; 87,6%), enquanto 28 (8,7%) foram diagnosticadas com carcinoma lobular e outros subtipos menos frequentes (carcinoma mucinoso, carcinoma metaplásico, carcinoma medular e carcinoma micropapilífero). O subtipo molecular luminal B foi predominante (140; 44,2%), enquanto o triplo negativo foi o menos comum (50; 15,8%).

A análise das características diagnósticas em relação aos subtipos histológicos, conforme apresentada na Tabela 1, não alcançou significância estatística.

**Tabela 1 -:** Relação entre características das mulheres adultas e idosas relacionadas ao câncer de mama e os subtipos histológicos de câncer de mama. São Paulo, SP, Brasil, 2023

**Idade no diagnóstico (anos)**		**Subtipo histológico**				**Total n=323**	**p-valor**
		**Carcinoma ductal**	**Carcinoma lobular**		**Outros**		
<40	n	73	3		2	78	0,253*
	%	93,6	3,8		2,6	100,0	
	Resíduos	1,8	-1,7		-0,6		
40 a 59	n	178	19		9	206	0,253*
	%	86,4	9,2		4,4	100,0	
	Resíduos	-0,9	0,5		0,8		
60+	n	32	6		1	39	0,253*
	%	82,1	15,4		2,6	100,0	
	Resíduos	-1,1	1,6		-0,4		
**IMC** ^†^						n=323	
<25,0	n	95	10		5	110	0,652*
	%	86,4	9,1		4,5	100,0	
	Resíduos	-0,5	0,2		0,6		
25,0 a 29,9	n	113	8		5	126	0,652*
	%	89,7	6,3		4,0	100,0	
	Resíduos	0,9	-1,2		0,2		
>=30	n	75	10		2	87	0,652*
	%	86,2	11,5		2,3	100,0	
	Resíduos	-0,5	1,1			-0,8	
**Total de familiares com câncer de mama**						n=323	
0	n	172	18		9	199	0,609*
	%	86,4	9,0		4,5	100,0	
	Resíduos	-0,8	0,3		1		
1	n	73	5		3	81	0,609*
	%	90,1	6,2		3,7	100,0	
	Resíduos	0,8	-0,9		0		
2+	n	38	5		0	43	0,609*
	%	88,4	11,6		0,0	100,0	
	Resíduos	0,2	0,7		-1,4		

*Teste Exato de Fisher; ^†^IMC = Índice de massa corporal

Conforme apresentado na Tabela 2, a análise das possíveis relações entre os hábitos de vida das participantes e os subtipos histológicos de câncer de mama apresentou diferença estatisticamente significativa entre o consumo diário de alimentos industrializados e os subtipos histológicos de câncer de mama (p=0,003). Dos participantes que consumiam alimentos industrializados diariamente, a maioria (243; 89,7%) apresentou carcinoma ductal. O consumo de refrigerantes ou sucos artificiais também mostrou significância estatística (p=0,044) em relação aos subtipos histológicos do câncer de mama.

**Tabela 2 -:** Relação entre hábitos de vida e os subtipos histológicos de câncer de mama apresentados em mulheres adultas e idosas. São Paulo, SP, Brasil, 2023

		**Subtipo histológico**			**Total**	**p-valor**
		**Carcinoma ductal**	**Carcinoma lobular**	**Outros**		
**Consumo de bebida alcoólica**					n=323	
Não	n	261	26	10	297	0,454*
	%	87,9	8,8	3,4	100,0	
	Resíduos	0,5	0,2	-1,1		
Sim	n	22	2	2	26	0,454*
	%	84,6	7,7	7,7	100,0	
	Resíduos	-0,5	-0,2	1,1		
**Consumo de tabaco**					n=323	
Nunca	n	172	16	10	198	0,554*
	%	86,9	8,1	5,1	100,0	
	Resíduos	-0,5	-0,5	1,6		
Ex fumante	n	75	7	1	83	0,554*
	%	90,4	8,4	1,2	100,0	
	Resíduos	0,9	-0,1	-1,4		
Fumante	n	36	5	1	42	0,554*
	%	85,7	11,9	2,4	100,0	
	Resíduos	-0,4	0,8	-0,5		
**Tempo de tabagismo (anos)**					n=125	
1 a 9	n	28	3	1	32	0,648*
	%	87,5	9,4	3,1	100,0	
	Resíduos	-0,3	-0,1	0,8		
10 a 19	n	26	1	0	27	0,648*
	%	96,3	3,7	0,0	100,0	
	Resíduos	1,4	-1,2	-0,7		
20 ou mais	n	57	8	1	66	0,648*
	%	86,4	12,1	1,5	100,0	
	Resíduos	-0,9	1	-0,1		
**Atividade física (minutos por semana)**					n=323	
Insuficientemente ativa (<150)	n	104	11	5	281	1,000*
	%	86,7	9,2	4,2	100,0	
	Resíduos	-0,6	0,4	0,5		
Suficientemente ativa (>=150)	n	38	3	1	42	1,000*
	%	90,5	7,1	2,4	100,0	
	Resíduos	0,6	-0,4	-0,5		
**Tipo de alimentação predominante ao longo da vida**						
**Alimentos industrializados (ingesta diária)**					n=323	
Não	n	40	6	6	52	0,003
	%	76,9	11,5	11,5	100,0	
	Resíduos	-2,6	0,8	3,3		
Sim	n	243	22	6	271	0,003
	%	89,7	8,1	2,2	100,0	
	Resíduos	2,6	-0,8	-3,3		
**Açúcar (ingesta diária)**					n=323	
Não	n	62	8	3	73	0,694*
	%	84,9	11,0	4,1	100,0	
	Resíduos	-0,8	0,8	0,2		
Sim	n	221	20	9	250	0,694*
	%	88,4	8,0	3,6	100,0	
	Resíduos	0,8	-0,8	-0,2		
**Leites e derivados do leite (ingesta diária)**					n=323	
Não	n	67	7	3	77	0,952*
	%	87,0	9,1	3,9	100,0	
	Resíduos	-0,2	0,2	0,1		
Sim	n	216	21	9	246	0,952*
	%	87,8	8,5	3,7	100,0	
	Resíduos	0,2	-0,2	-0,1		
**Legumes e frutas (ingesta diária)**					n=323	
Não	n	30	3	0	33	0,752*
	%	90,9	9,1	0,0	100,0	
	Resíduos	0,6	0,1	-1,2		
Sim	n	253	25	12	290	0,752*
	%	87,2	8,6	4,1	100,0	
	Resíduos	-0,6	-0,1	1,2		
**Refrigerantes ou suco artificial (frequência semanal)**					n=322*	
0	n	103	15	8	126	0,044
	%	81,7	11,9	6,3	100,0	
	Resíduos	-2,5	1,6	2		
1 a 2	n	89	8	0	97	0,044
	%	91,8	8,2	0,0	100,0	
	Resíduos	1,5	-0,2	-2,3		
3+	n	90	5	4	99	0,044
	%	90,9	5,1	4,0	100,0	
	Resíduos	1,2	-1,5	0,2		

*Teste Exato de Fisher

A análise da relação entre características relacionadas ao câncer de mama e os subtipos moleculares de tumores apresentados pelas mulheres, apresentada na Tabela 3, evidenciou uma diferença estatisticamente significativa entre a idade no momento do diagnóstico e os subtipos moleculares de câncer de mama (p=0,006). Mulheres com idade inferior a 40 anos apresentaram predominantemente os subtipos luminal B e TN, enquanto aquelas com idades entre 40 e 59 anos mostraram maior prevalência dos subtipos luminal B e luminal A.

**Tabela 3 -:** Relação entre características relacionadas ao câncer de mama e os subtipos moleculares de câncer de mama apresentados pelas mulheres participantes do estudo. São Paulo, SP, Brasil, 2023

		Subtipo molecular*				**Total**	**p-valor**
		**Luminal A**	**Luminal B**	**HER 2+**	**Triplo negativo**		
**Idade no diagnóstico (anos)**						n=317	
<40	n	13	29	16	20	78	0,006
	%	16,7	37,2	20,5	25,6	100,0	
	Resíduos	-1,7	-1,3	0,9	2,8		
40 a 59	n	46	94	34	28	202	0,006
	%	22,8	46,5	16,8	13,9	100,0	
	Resíduos	-0,5	1,4	-0,1	-1,2		
60+	n	16	15	4	2	37	0,006
	%	43,2	40,5	10,8	5,4	100,0	
	Resíduos	3	-0,4	-1,1	-1,8		
**IMC** ^†^						n=317	
<25,0	n	27	48	14	17	106	0,389 ^‡^
	%	25,5	45,3	13,2	16,0	100,0	
	Resíduos	0,5	0,4	-1,3	0,1		
25,0 a 29,9	n	32	49	27	16	124	0,389 ^‡^
	%	25,8	39,5	21,8	12,9	100,0	
	Resíduos	0,7	-1,2	1,8	-1,1		
>=30	n	16	41	13	17	87	0,389 ^‡^
	%	18,4	47,1	14,9	19,5	100,0	
	Resíduos	-1,4	0,8	-0,6	1,1		
**Familiares com câncer de mama**						n=317	
0	n	41	84	35	36	196	0,048 ^‡^
	%	20,9	42,9	17,9	18,4	100,0	
	Resíduos	-1,5	-0,3	0,5	1,6		
1	n	22	39	13	4	78	0,048 ^‡^
	%	28,2	50,0	16,7	5,1	100,0	
	Resíduos	1,1	1,3	-0,1	-3		
2+	n	12	15	6	10	43	0,048 ^‡^
	%	27,9	34,9	14,0	23,3	100,0	
	Resíduos	0,7	-1,2	-0,6	1,4		

*Prontuário sem dados da imuno-histoquímica de 6 mulheres; ^†^IMC = Índice de massa corporal; ^‡^Teste Exato de Fisher

Conforme apresentado na Tabela 4, não foram encontradas associações estatisticamente significativas entre os subtipos moleculares de câncer de mama e hábitos de vida das mulheres investigadas.

**Tabela 4 -:** Relação entre hábitos de vida e os subtipos moleculares de câncer de mama apresentados pelas mulheres participantes do estudo. São Paulo, SP, Brasil, 2023

		Subtipo molecular*				**Total**	**p-valor**
		**Luminal A**	**Luminal B**	**HER 2+**	**Triplo negativo**		
**Consumo de bebida alcoólica**						n=317	
Não	n	66	127	53	46	292	0,189 ^†^
	%	22,6	43,5	18,2	15,8	100,0	
	Resíduos	-1,5	0	1,8	0		
Sim	n	9	11	1	4	25	0,189 ^†^
	%	36,0	44,0	4,0	16,0	100,0	
	Resíduos	1,5	0	-1,8	0		
**Consumo de tabaco**						n=317	
Nunca	n	42	85	33	35	195	0,695 ^†^
	%	21,5	43,6	16,9	17,9	100,0	
	Resíduos	-1,1	0	-0,1	1,3		
Ex fumante	n	22	37	14	8	81	0,695 ^†^
	%	27,2	45,7	17,3	9,9	100,0	
	Resíduos	0,9	0,5	0,1	-1,7		
Fumante	n	11	16	7	7	41	0,695 ^†^
	%	26,8	39	17,1	17,1	100,0	
	Resíduos	0,5	-0,6	0	0,2		
**Tempo de tabagismo (anos)**						n=122	
1 a 9	n	6	20	3	3	32	0,290 ^†^
	%	18,8	62,5	9,4	9,4	100,0	
	Resíduos	-1,2	2,5	-1,4	-0,6		
10 a 19	n	7	11	4	4	26	0,290 ^†^
	%	26,9	42,3	15,4	15,4	100,0	
	Resíduos	0	-0,1	-0,3	0,5		
20 ou mais	n	20	22	14	8	64	0,290 ^†^
	%	31,3	34,4	21,9	12,5	100,0	
	Resíduos	1,1	-2,1	1,4	0,1		
**Atividade física (minutos por semana)**						n=317	
Insuficientemente ativa (<150)	n	65	118	48	46	277	0,646 ^†^
	%	28,8	40,7	13,6	16,9	100,0	
	Resíduos	0,5	-1	-0,2	1,1		
Suficientemente ativa (>=150)	n	10	20	6	4	40	0,646 ^†^
	%	25	50	15	10	100,0	
	Resíduos	-0,5	1	0,2	-1,1		
**Tipo de alimentação predominante ao longo da vida**							
**Alimentos industrializados (ingesta diária)**							
Não	n	13	23	8	8	52	0,992 ^†^
	%	25	44,2	15,4	15,4	100,0	
	Resíduos	0,2	0,1	-0,3	-0,1		
Sim	n	62	115	46	42	265	0,992 ^†^
	%	23,4	43,4	17,4	15,8	100,0	
	Resíduos	-0,2	-0,1	0,3	0,1		
**Açúcar (ingesta diária)**						n=317	
Não	n	22	33	6	9	70	0,072 ^†^
	%	31,4	47,1	8,6	12,9	100,0	
	Resíduos	1,7	0,7	-2,1	-0,8		
Sim	n	53	105	48	41	247	0,072 ^†^
	%	21,5	42,5	19,4	16,6	100,0	
	Resíduos	-1,7	-0,7	2,1	0,8		
**Leites e derivados do leite (ingesta diária)**						n=317	
Não	n	18	35	10	14	77	0,690 ^†^
	%	23,4	45,5	13,0	18,2	100,0	
	Resíduos	-0,1	0,4	-1,1	0,7	-0,1	
Sim	n	57	103	44	36	240	0,690 ^†^
	%	23,8	42,9	18,3	15,0	100,0	
	Resíduos	0,1	0,4	1,1	0,7		
**Legumes e frutas (ingesta diária)**						n=317	
Não	n	6	13	7	7	33	0,607 ^†^
	%	18,2	39,4	21,2	21,2	100,0	
	Resíduos	-0,8	-0,5	0,7	0,9		
Sim	n	69	125	47	43	284	0,607 ^†^
	%	24,3	44,0	16,5	15,1	100,0	
	Resíduos	0,8	0,5	-0,7	-0,9		
**Refrigerantes ou suco artificial (frequência semanal)**						n=316 ^‡^	
0	n	36	53	16	19	124	0,451 ^†^
	%	29,0	42,7	12,9	15,3	100,0	
	Resíduos	1,8	-0,3	-1,5	-0,2	93	
1 a 2	n	17	40	21	15	100,0	0,451 ^†^
	%	18,3	43,0	22,6	16,1		
	Resíduos	-1,5	-0,2	1,8	0,1	99	
3+	n	22	45	16	16	100,0	0,451 ^†^
	%	22,2	45,5	16,2	16,2		
	Resíduos	-0,4	0,4				

*Prontuário sem dados da imuno-histoquímica de 6 mulheres; ^†^Teste Exato de Fisher; ^‡^Sem resposta de 1 mulher

## Discussão

A análise dos dados obtidos neste estudo, envolvendo 323 mulheres, revelou associações importantes entre as características demográficas, hábitos alimentares e os subtipos de câncer de mama. Observamos que o não consumo de alimentos industrializados, assim como o não consumo de refrigerantes ou sucos artificiais, esteve associado a uma menor proporção de carcinoma ductal. Além disso, mulheres com menos de 40 anos apresentaram uma maior ocorrência do subtipo de câncer de mama triplo-negativo. Esses achados reforçam a influência da idade no perfil molecular do câncer de mama e destacam a relevância de fatores dietéticos nos diferentes subtipos histológicos, sublinhando a necessidade de integrar essas variáveis ao desenvolver estratégias de prevenção e políticas de saúde eficazes para o manejo da doença.

Entretanto, ao interpretar esses resultados, é essencial considerar as limitações do estudo. A coleta de informações, baseada em autorrelatos, apresenta vulnerabilidades como imprecisões de aferição e possíveis vieses de memória, os quais podem variar conforme a faixa etária das entrevistadas. Além disso, a representatividade da amostra deve ser vista com cautela, pois ela pode não refletir completamente a população geral, dificultando a generalização dos resultados. O viés de sobrevivência é outra preocupação relevante, uma vez que a inclusão exclusiva de mulheres diagnosticadas e tratadas pode distorcer a relação observada entre hábitos de vida, características diagnósticas e ocorrência de subtipos de câncer de mama. Isso pode levar à superestimação das associações, pois casos com desfechos desfavoráveis podem estar sub-representados.

A coleta de dados também foi afetada por fatores externos, como a pandemia causada pelo vírus SARS-CoV. A necessidade de adaptar os procedimentos de coleta, com a submissão de alterações aos comitês de ética em pesquisa (CEP) da Escola de Enfermagem e da Fundação Faculdade de Medicina da USP, gerou atrasos e eliminou a possibilidade de realizar entrevistas presenciais. Essa modificação limitou o contato direto entre pesquisador e participantes, potencialmente influenciando a profundidade e a qualidade das informações coletadas.

Mesmo diante dessas limitações, os achados sobre a distribuição dos tipos de câncer de mama são consistentes com dados populacionais. A maioria (87,6%) dos casos foi de carcinoma ductal, um resultado alinhado à prevalência geral desse tipo na população^(13)^. Outros subtipos histológicos, como carcinoma mucinoso, carcinoma metaplásico, carcinoma medular e carcinoma micropapilífero também foram identificados, embora raros, cada um representando menos de 5% dos casos, conforme descrito na literatura^(4)^.

No que tange aos subtipos moleculares, a predominância dos tumores luminais em nosso estudo reforça a evidência global de que eles correspondem a cerca de 80% dos casos de câncer de mama, enquanto o subtipo triplo-negativo (TN) abrange aproximadamente 10-15% dos diagnósticos^(14)^. Essa concordância com dados internacionais fortalece a validade de nossos resultados, indicando que o perfil molecular observado corrobora com a tendência já documentada na literatura.

Ademais, a análise da variável “idade no momento do diagnóstico” revelou que a maioria das participantes do estudo estava na faixa etária de 40 a 59 anos. De acordo com o INCA, a maior concentração de exames de rastreamento, como a mamografia, ocorre entre mulheres de 50 a 59 anos, seguida da faixa etária de 60 a 69 anos^(3)^. Em contraste, a ACS recomenda o início do rastreamento a partir dos 40 anos^(4)^. Essas diferenças nas recomendações sublinham a importância de uma discussão contínua sobre a melhor idade para iniciar o rastreamento, levando em conta o contexto epidemiológico e as especificidades da população estudada.

Nosso estudo identificou uma associação significativa entre a idade e os subtipos moleculares do câncer de mama, com um aumento do risco de câncer triplo-negativo (TN) em mulheres com menos de 40 anos. Essa observação é corroborada por um registro prospectivo no Brasil, que indicou uma maior proporção de tumores Luminal B e TN em pacientes mais jovens^(15)^. Além disso, a literatura já estabelece que os cânceres TN são diagnosticados de forma desproporcional em mulheres mais jovens^(16)^.

Esses achados destacam a importância de incorporar a diversidade dos subtipos moleculares e a variabilidade no risco entre diferentes faixas etárias, ao elaborar diretrizes de rastreamento. Assim, torna-se essencial aprofundar as investigações sobre qual seria a idade mais adequada para o início do rastreamento, garantindo uma detecção precoce e eficaz em diferentes grupos populacionais.

Adicionalmente, 39% das mulheres deste estudo apresentaram um índice de massa corporal (IMC) entre 25 e 29,9 kg/m², enquanto o relatório de 2023 do Sistema de Vigilância Alimentar e Nutricional identificou que 33,7% da população adulta feminina brasileira está com sobrepeso^(17)^. Apesar disso, a análise não revelou uma associação estatisticamente significativa entre o IMC e os subtipos histológicos ou moleculares do câncer de mama. Esses resultados estão em consonância com outro estudo que, embora reconheça a relação entre IMC elevado e o risco geral de câncer de mama, não encontrou significância ao examinar essa relação com subtipos específicos da doença^(18)^. Ressalta-se que a literatura ainda carece de estudos mais detalhados sobre essas associações.

No que tange a fatores como consumo de bebida alcoólica, tabagismo e prática de atividade física, não identificamos associações estatisticamente significativas em nosso estudo, diferindo do que é amplamente documentado. Por exemplo, uma análise de uma coorte prospectiva americana demonstrou uma correlação significativa entre o consumo de álcool e o aumento do risco de tumores com receptor hormonal positivo, sobretudo carcinoma lobular, com efeitos mais pronunciados em mulheres pós-menopáusicas^(19)^. Esses achados vão de encontro à ideia da influência de mecanismos hormonais na relação entre o álcool e o câncer de mama, destacando o papel modulador do estrogênio^(19)^.

Quanto ao tabagismo, uma meta-análise abrangente, envolvendo dados de 14 estudos de coorte, revelou um aumento geral modesto (7%) no risco de câncer de mama entre mulheres que fumavam na época da pesquisa^(20)^. Essa associação foi particularmente relevante em cânceres que expressam o receptor de estrogênio (RE), com um risco aumentado de 76% para tumores RE positivos^(20)^. Ademais, o impacto do tabagismo parece variar conforme a idade de início. Mulheres que começaram a fumar antes da primeira gravidez apresentaram um risco ainda mais elevado de desenvolver câncer de mama^(20)^.

Esses resultados ressaltam a complexidade das interações entre fatores de estilo de vida e o risco de câncer de mama, sugerindo que a idade e o momento da exposição a fatores como o tabaco desempenham papéis fundamentais na carcinogênese mamária. Portanto, investigações futuras são necessárias para entender melhor essas dinâmicas e informar estratégias preventivas personalizadas.

Contrariando os achados do presente estudo, que não encontrou significância estatística na relação entre a prática de atividade física e os subtipos histológicos ou moleculares do câncer de mama, um estudo caso-controle realizado na Alemanha demonstrou uma associação inversa significativa entre a prática de atividade física e o risco de câncer de mama na fase pós-menopausa^(21)^. Os autores relataram que o efeito protetor foi mais pronunciado em carcinomas invasivos com receptor hormonal positivo^(21)^. Além disso, verificou-se que a atividade física realizada após os 50 anos se mostrou mais eficaz na prevenção de carcinomas RE+ e RP+ em mulheres na pós-menopausa do que a prática em idades mais jovens^(21)^.

No âmbito do presente estudo, conseguimos estabelecer significância estatística nas relações entre os subtipos histológicos e algumas variáveis relacionadas aos hábitos alimentares, especificamente no que tange à ingestão de alimentos industrializados (p=0,003) e o consumo de refrigerantes ou sucos artificiais (p=0,044) (Tabela 2). Em relação à dieta, a literatura científica apresenta resultados heterogêneos. Alguns estudos sugerem que a enzima aromatase, presente no tecido adiposo, pode converter colesterol circulante em estradiol, aumentando os níveis desse hormônio nos tecidos mamários e influenciando a expressão dos receptores de estrogênio, o que pode afetar o comportamento das células cancerígenas^(22)^. Além disso, estudos revelam que dietas ricas em alimentos curados podem estar associadas a um risco aumentado de câncer de mama receptor hormonal negativo, enquanto o consumo de alimentos grelhados se correlaciona com um risco maior de câncer de mama receptor hormonal positivo^(23)^.

O papel dos alimentos industrializados também merece destaque. Esses alimentos, incluindo os processados e embutidos, contribuem para uma dieta pró-inflamatória, enquanto uma alimentação rica em frutas, vegetais e grãos integrais está associada a menores níveis de biomarcadores inflamatórios^(24)^. Por outro lado, o consumo elevado de carne vermelha e processada está relacionado a um aumento desses marcadores inflamatórios^(24)^. Neste estudo, observamos diferenças significativas no consumo de alimentos industrializados associadas ao carcinoma ductal. Especificamente, a frequência de carcinoma ductal foi menor entre mulheres que relataram abster-se de alimentos industrializados e de refrigerantes ou sucos artificiais, com valores de resíduos de -2,6 e -2,5, respectivamente (p=0,044). Isso sugere que a ausência de consumo desses produtos pode estar associada a uma menor incidência de carcinoma ductal.

No entanto, não encontramos significância estatística ao analisar as associações entre o consumo de alimentos industrializados, refrigerantes ou sucos artificiais e os subtipos moleculares. Embora outros estudos tenham relatado uma relação entre dietas pró-inflamatórias e subtipos de câncer de mama positivos para receptor de estrogênio ou de progesterona, bem como para o receptor HER2, essa associação não foi observada no caso do subtipo triplo-negativo (TN)^(24)^.

É importante salientar que a literatura ainda é escassa no que diz respeito à caracterização dos subtipos histológicos do câncer de mama, com maior ênfase sendo dada aos subtipos moleculares. Embora este estudo seja de natureza transversal, o que impede a inferência de causalidade, nossos achados fornecem evidências significativas que contribuem para a geração de hipóteses e a identificação de fatores de risco ou marcadores que merecem investigações mais aprofundadas. Estudos transversais, como este, são fundamentais para levantar hipóteses e sugerir direções para pesquisas futuras, especialmente por meio de estudos longitudinais ou experimentais, que poderiam esclarecer as relações causais.

Em resumo, mesmo que as associações observadas não estabeleçam uma relação direta de causa e efeito, os resultados sugerem conexões relevantes entre os hábitos de vida, características diagnósticas e os subtipos de câncer de mama. Esses achados têm o potencial de orientar pesquisas futuras e contribuir significativamente para o avanço do conhecimento na área. Além disso, nossos resultados oferecem suporte valioso para a prática da Enfermagem, ressaltando a importância de promover estilos de vida saudáveis e prestar assistência qualificada às mulheres em tratamento ou acompanhamento por câncer de mama. Destacamos a necessidade de estudos adicionais, especialmente na população brasileira, para desenvolver estratégias preventivas eficazes contra a doença.

## Conclusão

Este estudo buscou investigar a complexa relação entre as características das mulheres relacionadas ao câncer de mama, seus hábitos de vida e a incidência de diferentes subtipos da doença. Os resultados obtidos contribuem significativamente para a compreensão dessas interações e sua influência na manifestação dos subtipos de câncer de mama.

Verificou-se uma associação significativa entre a idade das mulheres no momento do diagnóstico e os subtipos moleculares do câncer de mama, determinados por imuno-histoquímica. Especificamente, mulheres com idade inferior a 40 anos apresentaram maior frequência para desenvolver o câncer de mama triplo negativo (TN).

Nossas descobertas destacam associações entre hábitos de vida, como o consumo de alimentos industrializados e refrigerantes ou sucos artificiais, e os subtipos histológicos de câncer de mama. Isso ressalta a importância de considerar tais fatores na prevenção da doença.

O presente estudo contribuiu substancialmente para os campos do ensino, pesquisa e assistência de Enfermagem, ao conferir maior visibilidade aos hábitos de vida modificáveis e identificados como fatores de risco para o câncer de mama. Além disso, a pesquisa fornece informações para orientar estratégias de saúde pública de prevenção, diagnóstico precoce e implementação de políticas eficazes para mitigar o impacto do câncer de mama na sociedade.

# Translation: Relación entre características diagnósticas, hábitos de vida y subtipos de cáncer de mama en mujeres^*^

**DOI:** 10.1590/1518-8345.7492.4580

**Authors:** Fernanda Cristina Marin, Clóvis Arlindo de Sousa, Erika de Sá Vieira Abuchaim, Isília Aparecida Silva

**Affiliations:** 1 Universidade de São Paulo, Escola de Enfermagem, São Paulo, SP, Brasil.; 2 Universidade Regional de Blumenau, Departamento de Educação Física e Desportos, Blumenau, SC, Brasil.; 3 Universidade Federal de São Paulo, Escola Paulista de Enfermagem, São Paulo, SP, Brasil.

**Keywords:** Neoplasias de la Mama, Hábitos, Dieta, Consumo de Bebidas Alcohólicas, Tabaquismo, Ejercicio Físico

## Abstract

investigar la relación entre características diagnósticas, hábitos de vida y la aparición de subtipos de cáncer de mama en mujeres adultas y mayores.

estudio transversal realizado con mujeres ≥ 18 años en tratamiento o seguimiento de neoplasia mamaria en un Instituto de Cáncer del estado de São Paulo. Los datos fueron recolectados entre diciembre/2022 y julio/2023 mediante entrevista telefónica y revisión de historiales electrónicos. El análisis estadístico incluyó distribución de frecuencia absoluta y relativa, y análisis bivariado, utilizando la prueba de Chi-Cuadrado o la prueba Exacta de Fisher, considerando valores estadísticamente significativos con p≤0,05, con un intervalo de confianza del 95%.

participaron en el estudio 323 mujeres. Las mujeres que no consumían alimentos industrializados, refrescos o jugos artificiales presentaron una menor proporción de carcinoma ductal. Y aquellas con edad < 40 años presentaron una mayor proporción de ocurrencia del subtipo de cáncer de mama triple negativo.

los resultados indican una relación significativa entre la edad al diagnóstico y el subtipo molecular, además de patrones relevantes entre el consumo de alimentos industrializados y refrescos o jugos artificiales y los subtipos histológicos de la enfermedad. Estos hallazgos resaltan la importancia de considerar tales factores en la prevención de la enfermedad.

## Destacados

**(1)** Relación significativa entre la edad al diagnóstico y el subtipo molecular. **(2)** Relación significativa entre el consumo de alimentos industrializados y subtipos histológicos. **(3)** Relación significativa entre el consumo de refrescos o jugos y subtipos histológicos. **(4)** Orientar estrategias de salud pública y prevención del cáncer de mama.

## Introducción

Según la Organización Mundial de la Salud (OMS), en 2020, un total de 2,3 millones de mujeres fueron diagnosticadas con cáncer de mama y, en el mismo período, 685.000 murieron en todo el mundo por neoplasia mamaria^(1)^. El *Global Cancer Observatory* (*Globocan*) corrobora la gravedad de este escenario al estimar que el 23,8% de los 20 millones de nuevos casos de cáncer registrados mundialmente correspondían a cáncer de mama en mujeres^(2)^.

En Brasil, el *Instituto Nacional de Câncer* (INCA) estima que en el trienio 2023-2025 se registrarán 704.000 nuevos casos de cáncer por año, siendo el cáncer de mama en mujeres responsable de 73.610 de ellos^(3)^. Estos datos resaltan la necesidad de acciones eficaces para el control y la prevención de esta neoplasia en la población femenina.

Desde el punto de vista histológico, el cáncer de mama puede clasificarse como carcinoma ductal (originado en los conductos mamarios) o carcinoma lobulillar (localizado en los lóbulos productores de leche)^(4)^. Además de estos, la *American Cancer Society* (ACS) reconoce otros cuatro subtipos especiales de neoplasia mamaria: carcinoma inflamatorio (caracterizado por signos inflamatorios en la piel), angiosarcoma mamario (subtipo raro que se inicia en las células de revestimiento de los vasos sanguíneos o linfáticos), tumor fibroide (desarrollado en el tejido conjuntivo de la mama) y enfermedad de Paget (comienza en los conductos mamarios y se extiende a la piel del pezón y la areola)^(4)^.

Los subtipos moleculares del cáncer de mama, categorizados a partir de características genéticas distintas, incluyen los subtipos: luminales (con receptores hormonales para estrógeno y progesterona positivos), HER-2 enriquecido (receptor del factor de crecimiento epidérmico humano 2 - HER2) y los basales (negativos para los receptores de estrógeno, progesterona y HER2), también conocidos como triple negativos (TNs)^(4)^. Los subtipos luminales fueron definidos por la 12.ª Conferencia de St. Gallen como luminal A, caracterizado por ser positivo para receptores de estrógeno (RE) y/o progesterona (RP), negativo para HER2, y con un bajo índice de proliferación celular (Ki-67<14%)^(5)^. El subtipo luminal B incluye casos que son positivos para RE y/o RP y negativos para HER2, pero con un alto índice de Ki-67, o que son positivos para RE y/o RP y HER2, independientemente del índice de Ki-67^(5)^.

Según la ACS, el subtipo luminal A es responsable de aproximadamente el 68% de todos los casos de cáncer de mama^(6)^. Por su parte, los subtipos luminal B y el triple negativo corresponden cada uno a aproximadamente el 10% de los casos^(6)^. Es importante destacar que el subtipo triple negativo presenta una incidencia de aproximadamente el 20% entre mujeres afroamericanas, siendo más prevalente en mujeres jóvenes y en aquellas portadoras de mutaciones del gen BRCA1. Por último, el HER2 enriquecido es el subtipo menos común, representando aproximadamente el 4% de todos los diagnósticos de cáncer de mama^(6)^.

Considerando esta distribución de los subtipos de cáncer de mama, un estudio realizado con 137 mujeres jóvenes (media de edad de 34,7 años), diagnosticadas con neoplasia mamaria reveló que el 11,4% (15) se encontraban en estadio cuatro en el momento del diagnóstico. Entre estas pacientes, el carcinoma ductal invasivo fue el tipo histológico predominante, identificado en el 86% (118) de los casos. La distribución de los subtipos moleculares identificados fue del 16,8% para el luminal A, 38,7% para el luminal B, 30,5% para el HER2 enriquecido y 15,3% para el triple negativo, reflejando la variación en la incidencia de los subtipos en una población joven^(7)^.

En lo que se refiere a los principales factores de riesgo asociados al cáncer de mama, el *National Cancer Institute* (NIH) destaca que los factores ambientales, incluidos los hábitos de vida, pueden influir tanto en el desarrollo como en la prevención de este^(8)^. Según el NIH, la relación entre los factores dietéticos y el cáncer es compleja y a menudo inconclusa, citando como ejemplo el hecho de que las dietas ricas en frutas, vegetales y calcio están asociadas a un menor riesgo de cáncer, aunque no exista un consenso definitivo al respecto^(8)^. Un estudio de revisión sistemática encontró una asociación débil entre el consumo de carne roja no procesada y un aumento del 6% en el riesgo de cáncer colorrectal, 3% en el cáncer de mama^(9)^.

El NIH destaca, además, que el consumo de alcohol está asociado a un aumento del riesgo de varios tipos de cáncer, incluyendo el cáncer de mama, hígado y colorrectal en mujeres, y que el tabaquismo es un posible factor de riesgo para el cáncer de mama, aumentando ligeramente el riesgo de diagnóstico, especialmente en fumadoras jóvenes con alto consumo, siendo la exposición al humo pasivo un factor de riesgo adicional, particularmente en la infancia y para el cáncer de mama premenopáusico^(8)^.

Por otro lado, la práctica regular de actividad física está asociada a una reducción del 10% al 20% en el riesgo de cáncer de mama, siendo inversamente proporcional e independiente del índice de masa corporal (IMC)^(8)^. No obstante, la obesidad es reconocida como un factor de riesgo para diversos tipos de cáncer, incluido el cáncer de mama en la postmenopausia^(10)^. Un estudio publicado en 2021 sugiere una fuerte asociación positiva entre un IMC elevado y los subtipos de cáncer de mama ER/PR+HER2− y HER2+ en mujeres postmenopáusicas en comparación con aquellas en premenopausia^(11)^. De acuerdo con la ACS, el riesgo de cáncer de mama receptor hormonal positivo en la postmenopausia es de 1,5 a 2 veces mayor en mujeres con sobrepeso u obesidad, posiblemente debido a niveles más altos de estrógeno provenientes del tejido adiposo y otros factores, como niveles elevados de insulina^(6)^.

Las posibles asociaciones entre los hábitos de vida y los diferentes subtipos de cáncer de mama aún son poco investigadas y, en este contexto, el presente estudio tiene como objetivo analizar la prevalencia y verificar las relaciones entre los hábitos de vida, las características diagnósticas y la ocurrencia de diferentes subtipos de cáncer de mama en mujeres adultas y de edad avanzada.

## Método

### Tipo de estudio

Se trata de un estudio transversal.

### Local

El estudio se realizó en el *Instituto do Câncer* del Estado de São Paulo (ICESP), ubicado en la región central de la capital paulista y parte del complejo del Hospital de Clínicas de la Facultad de Medicina de la *Universidade de São Paulo* (HCFMUSP).

### Período

La recolección de datos se realizó en el período de diciembre de 2020 a julio de 2023.

### Población

Participaron en el estudio mujeres de 18 años o más, diagnosticadas con cualquier subtipo de cáncer de mama, en tratamiento o seguimiento en la institución. No se establecieron restricciones respecto al tiempo transcurrido desde el diagnóstico ni a la edad máxima de las participantes. Como criterio de exclusión, se consideraron inelegibles a las pacientes que, en el momento de la recolección de datos, presentaban limitaciones físicas, cognitivas o emocionales que impidieran su participación en el estudio.

La selección de la muestra se basó en un informe del Sector de Gestión de Información en Salud (GIS) del ICESP, que proporcionó el nombre y el contacto telefónico de 1.881 mujeres atendidas en los sectores de quimioterapia, radioterapia, rehabilitación y consulta ambulatoria en el período de junio de 2019 a mayo de 2020.

El cálculo muestral se realizó con base en el tamaño de la muestra para frecuencia en una población, considerando el tamaño de la población (para el factor de corrección de la población finita) (N): 1.881; frecuencia % hipotética del factor del resultado en la población (p): 50%+/-5%; límites de confianza como % de 100 (absoluto+/-%) (d): 5%; y efecto de diseño (para encuestas en grupo-EDFF):1. El tamaño de la muestra calculado fue de 320 mujeres, con un intervalo de confianza del 95%.

### Instrumento de recolección de datos

El instrumento de recolección fue desarrollado específicamente para este estudio, estructurado en dos partes principales: la primera abarca características sociodemográficas y hábitos de vida, recogidas mediante entrevistas individuales realizadas por medio de contacto telefónico, y la segunda, características diagnósticas, obtenidas a partir de consulta a expedientes electrónicos. Para garantizar la validez de contenido y la precisión del instrumento, el equipo de investigación, compuesto por especialistas en lactancia y cáncer de mama, trabajó conjuntamente en la elaboración y revisión de las preguntas basándose en los resultados del test piloto. Este proceso colaborativo fue crucial para la construcción y revisión de las preguntas, asegurando su pertinencia y alineación con los objetivos de la investigación. Inicialmente elaborado en *Microsoft Word*, el instrumento fue transferido al *software REDCap*.

Tras la aprobación por parte de los Comités de Ética en Investigación (CEPs), se llevó a cabo un test piloto con el objetivo de evaluar la consistencia, aplicabilidad y eficacia del instrumento de recolección de datos. El proceso permitió verificar la claridad de las preguntas y la comprensión por parte de las mujeres, ajustar el formato de las entrevistas y estimar el tiempo medio necesario para la recolección. Además, posibilitó un flujo de comunicación que abarcó adecuadamente el tema para alcanzar los objetivos del estudio. Durante esta etapa, se realizaron ocho entrevistas, cuyos resultados indicaron la adecuación del instrumento y de la dinámica de comunicación, no siendo necesarias modificaciones. Las entrevistas fueron incorporadas a la investigación, demostrando la utilidad y solidez de esta etapa inicial.

El test piloto fue fundamental para confirmar la elaboración consistente y criteriosa del instrumento, demostrando la confiabilidad y validez del proceso de recolección de datos. Este procedimiento inicial no solo validó el instrumento, sino que aseguró que el cuestionario cumpliera con los objetivos del estudio.

### Variables

La caracterización de la muestra se realizó mediante las siguientes variables y categorizaciones: edad en el momento de la entrevista (<40 años, 40 a 59 años y 60+ años), raza autorreferida (amarilla, blanca, parda, negra, no sabe/no quiso informar), escolaridad (analfabeta, educación primaria, educación secundaria, educación superior y posgrado), estado civil (soltera, casada, unión estable, viuda, separada o divorciada, no quiso informar), región de nacimiento (Norte, Nordeste, Centro-Oeste, Sur, Sudeste), región donde vivió la mayor parte de la vida (Norte, Nordeste, Centro-Oeste, Sur, Sudeste) e ingresos familiares (categorizados en salario mínimo vigente en la época - R$ 1.039,00 - menos de dos, dos a cuatro, cinco o más).

Los hábitos de vida, considerados como variables independientes en el presente estudio, fueron: consumo de bebida alcohólica (sí o no), consumo de tabaco (nunca, exfumador o fumador), actividad física (insuficientemente activa - <150 min/semana o suficientemente activa - >=150 min/semana) y el tipo de alimentación predominante en la mayor parte de la vida.

Considerando también como variable independiente, el patrón alimentario predominante se evaluó mediante la investigación del consumo diario de diferentes tipos de alimentos: productos industrializados (embutidos, enlatados o procesados), azúcar, leches (y derivados), verduras (y frutas) y refresco o jugo artificial. Todos fueron categorizados individualmente como “sí” o “no”. En casos afirmativos, se detalló la frecuencia de la ingesta, clasificándola como diaria, semanal, mensual o rara.

Aún como variable independiente, las características diagnósticas incluyeron: edad al diagnóstico (<40 años, 40 a 59 años y 60+ años), índice de masa corporal (IMC) al diagnóstico (normal <25,0, sobrepeso 25,0 a 29,9 y obesa >=30 kg/m²) y antecedentes de cáncer en la familia (sí o no). En casos afirmativos, se detalló el tipo específico de cáncer y el grado de parentesco de los individuos afectados.

Como variable dependiente del estudio, el cáncer de mama se clasificó en subtipos histológicos (carcinoma ductal, carcinoma lobular u otros) o moleculares (luminal A, luminal B, HER2+ o TN).

Se consideró como desenlace la ocurrencia de los diferentes subtipos de cáncer de mama en relación con los hábitos de vida y las características diagnósticas de las participantes.

### Recolección de datos

La relación que contenía los nombres y teléfonos de las 1.881 mujeres entregada en el informe del ICESP se entregó una sola vez y se utilizó durante todo el procedimiento de recolección de datos. Semanalmente, siguiendo el orden alfabético de los nombres de esta lista, se seleccionaban 15 mujeres para ser invitadas a participar en el estudio, lo que se realizaba mediante llamada telefónica. Es relevante destacar que esta muestra (15 mujeres/semana) se ajustó de acuerdo con la cantidad de respuestas obtenidas, modificándose según fuera necesario.

La recolección de datos se realizó en dos momentos distintos: 1. Presentación del estudio y entrevista y 2. Consulta al expediente electrónico institucional de la paciente. Durante el contacto telefónico se presentó detalladamente el estudio, se leyó y grabó el Término de Consentimiento Libre e Informado. Las mujeres que aceptaban participar en el estudio eran entrevistadas por la investigadora principal o colaboradora (enfermera especializada en lactancia), capacitada para tal fin. Todas las entrevistas realizadas fueron grabadas y almacenadas en la computadora de la investigadora principal de manera segura, protegida con contraseña que solo ella conocía. El acceso a los expedientes electrónicos, para la extracción de los datos a utilizar en el estudio, se realizó exclusivamente por la investigadora dentro de la institución.

### Análisis de los datos

Se utilizó la base de datos de la plataforma *REDCap* para el registro de los datos, los cuales fueron organizados, tratados estadísticamente y analizados utilizando los recursos del programa *Statistical Package for the Social Sciences* – SPSS, versión 22.

Se realizó estadística descriptiva, con distribución de frecuencia absoluta y relativa para todas las variables, presentadas mediante tablas y gráficos. Para el análisis bivariado con cáncer de mama, se utilizó la prueba de Chi-Cuadrado o la prueba Exacta de Fisher, cuando más del 20% de las frecuencias esperadas fueron menores a cinco. Para todos los análisis estadísticos se adoptó un nivel de significancia de p≤0,05, correspondiente a un intervalo de confianza del 95%. La razón de prevalencia se utilizó como medida de efecto, proporcionando una cuantificación de las asociaciones entre los hábitos de vida y la incidencia de los diversos subtipos de cáncer de mama. Los residuos estandarizados ajustados con valores superiores a +2,0 o inferiores a –2,0 se consideraron para identificar diferencias estadísticas entre las categorías de la variable asociada.

### Aspectos éticos

Esta investigación siguió los preceptos éticos de investigaciones que involucran seres humanos, establecidos por la *Resolução* 466/12 del *Conselho Nacional de Saúde* ^(12)^.

De acuerdo con los requisitos vigentes, este proyecto fue sometido al Comité de Ética en Investigación de la *Escola de Enfermagem* de la USP (EEUSP) –dictamen 4.341.047. Pasó por los trámites de evaluación y autorización del CEP en el Hospital de Clínicas de la *Universidade de São Paulo* (CEP-HCFMUSP) – dictamen 4.303.589 – conforme a sus exigencias institucionales.

## Resultados

Los contactos telefónicos se realizaron conforme al diagrama de flujo de la Figura 1.

**Figura 1 -:** Diagrama de flujo de los contactos telefónicos

Así, el estudio incluyó a 323 mujeres con edades entre 26 y 86 años. La mayoría, 213 (65,9%), tenía entre 40 y 59 años y se autodeclaró blanca (174, 53,9%). La escolaridad predominante fue educación secundaria o superior (110, 34,1% y 114, 35,3%, respectivamente). De las mujeres entrevistadas, 139 (43%) estaban casadas, la mayoría (237, 73,4%) era originaria de la región Sudeste y 301 (93,2%) pasó la mayor parte de su vida en dicha región.

Los ingresos familiares de 69 (21,4%) de las mujeres eran inferiores a dos salarios mínimos, mientras que 150 (46,4%) recibían hasta cuatro salarios mínimos. En cuanto a los hábitos de vida, la mayoría, 297 (92%), no consumía alcohol y 198 (61,3%) nunca había fumado. Solo 42 (13%) practicaban actividad física regularmente. Respecto a la preferencia alimentaria a lo largo de la vida, 271 (83,9%) de las participantes reportaron el consumo diario de alimentos industrializados. Además, 250 (77,4%) refirieron ingerir azúcar diariamente, ya sea para endulzar bebidas como café y jugos o como parte integrante de alimentos dulces. Adicionalmente, 196 (60,9%) de las mujeres reportaron el consumo diario de refrescos o jugos artificiales.

En el momento del diagnóstico, la mayoría (206, 63,8%) tenía entre 40 y 59 años y 126 (39%) presentaban un índice de masa corporal entre 25 y 29,9. Además, 124 (38,4%) reportaron antecedentes familiares de cáncer de mama. El carcinoma ductal fue el tipo histológico más común (283, 87,6%), mientras que 28 (8,7%) fueron diagnosticadas con carcinoma lobulillar y otros subtipos menos frecuentes (carcinoma mucinoso, carcinoma metaplásico, carcinoma medular y carcinoma micropapilífero). El subtipo molecular luminal B fue predominante (140, 44,2%), mientras que el triple negativo fue el menos común (50, 15,8%).

El análisis de las características diagnósticas en relación con los subtipos histológicos, según se presenta en la Tabla 1, no alcanzó significación estadística.

**Tabla 1 -:** Relación entre las características de las mujeres adultas y de edad avanzada relacionadas con el cáncer de mama y los subtipos histológicos de cáncer de mama. São Paulo, SP, Brasil, 2023

**Edad al diagnóstico (años)**		**Subtipo histológico**			**Total n=323**	**p-valor**
		**Carcinoma ductal**	**Carcinoma lobular**	**Otros**		
<40	n	73	3	2	78	0,253*
	%	93,6	3,8	2,6	100,0	
	Residuos	1,8	-1,7	-0,6		
40 a 59	n	178	19	9	206	0,253*
	%	86,4	9,2	4,4	100,0	
	Residuos	-0,9	0,5	0,8		
60+	n	32	6	1	39	0,253*
	%	82,1	15,4	2,6	100,0	
	Residuos	-1,1	1,6	-0,4		
**IMC** ^†^					n=323	
<25,0	n	95	10	5	110	0,652*
	%	86,4	9,1	4,5	100,0	
	Residuos	-0,5	0,2	0,6		
25,0 a 29,9	n	113	8	5	126	0,652*
	%	89,7	6,3	4,0	100,0	
	Residuos	0,9	-1,2	0,2		
>=30	n	75	10	2	87	0,652*
	%	86,2	11,5	2,3	100,0	
	Residuos	-0,5	1,1	-0,8		
**Total de familiares con cáncer de mama**					n=323	
0	n	172	18	9	199	0,609*
	%	86,4	9,0	4,5	100,0	
	Residuos	-0,8	0,3	1		
1	n	73	5	3	81	0,609*
	%	90,1	6,2	3,7	100,0	
	Residuos	0,8	-0,9	0		
2+	n	38	5	0	43	0,609*
	%	88,4	11,6	0,0	100,0	
	Residuos	0,2	0,7	-1,4		

*Prueba Exacta de Fisher; ^†^IMC = Índice de masa corporal

Conforme a lo presentado en la Tabla 2, el análisis de las posibles relaciones entre los hábitos de vida de las participantes y los subtipos histológicos de cáncer de mama mostró una diferencia estadísticamente significativa entre el consumo diario de alimentos industrializados y los subtipos histológicos de cáncer de mama (p=0,003). De las participantes que consumían alimentos industrializados diariamente, la mayoría (243, 89,7%) presentó carcinoma ductal. El consumo de refrescos o jugos artificiales también mostró significancia estadística (p=0,044) en relación con los subtipos histológicos del cáncer de mama.

**Tabla 2 -:** Relación entre hábitos de vida y los subtipos histológicos de cáncer de mama presentados en mujeres adultas y de edad avanzada. São Paulo, SP, Brasil, 2023

		**Subtipo histológico**			**Total**	**p-valor**
		**Carcinoma ductal**	**Carcinoma lobular**	**Otros**		
**Consumo de bebida alcohólica**					n=323	
No	n	261	26	10	297	0,454*
	%	87,9	8,8	3,4	100,0	
	Residuos	0,5	0,2	-1,1		
Sí	n	22	2	2	26	0,454*
	%	84,6	7,7	7,7	100,0	
	Residuos	-0,5	-0,2	1,1		
**Consumo de tabaco**					n=323	
Nunca	n	172	16	10	198	0,554*
	%	86,9	8,1	5,1	100,0	
	Residuos	-0,5	-0,5	1,6		
Exfumante	n	75	7	1	83	0,554*
	%	90,4	8,4	1,2	100,0	
	Residuos	0,9	-0,1	-1,4		
Fumante	n	36	5	1	42	0,554*
	%	85,7	11,9	2,4	100,0	
	Residuos	-0,4	0,8	-0,5		
**Tiempo de tabaquismo (años)**					n=125	
1 a 9	n	28	3	1	32	0,648*
	%	87,5	9,4	3,1	100,0	
	Residuos	-0,3	-0,1	0,8		
10 a 19	n	26	1	0	27	0,648*
	%	96,3	3,7	0,0	100,0	
	Residuos	1,4	-1,2	-0,7		
20 o más	n	57	8	1	66	0,648*
	%	86,4	12,1	1,5	100,0	
	Residuos	-0,9	1	-0,1		
**Actividad física (minutos por semana)**					n=323	
Insuficientemente activa (<150)	n	104	11	5	281	1,000*
	%	86,7	9,2	4,2	100,0	
	Residuos	-0,6	0,4	0,5		
Suficientemente activa (>=150)	n	38	3	1	42	1,000*
	%	90,5	7,1	2,4	100,0	
	Residuos	0,6	-0,4	-0,5		
**Tipo de alimentación predominante a lo largo de la vida**						
**Alimentos industrializados (ingesta diaria)**					n=323	
No	n	40	6	6	52	0,003
	%	76,9	11,5	11,5	100,0	
	Residuos	-2,6	0,8	3,3		
Sí	n	243	22	6	271	0,003
	%	89,7	8,1	2,2	100,0	
	Residuos	2,6	-0,8	-3,3		
**Azúcar (ingesta diaria)**					n=323	
No	n	62	8	3	73	0,694*
	%	84,9	11,0	4,1	100,0	
	Residuos	-0,8	0,8	0,2		
Sí	n	221	20	9	250	0,694*
	%	88,4	8,0	3,6	100,0	
	Residuos	0,8	-0,8	-0,2		
**Leches y derivados de la leche (ingesta diaria)**					n=323	
No	n	67	7	3	77	0,952*
	%	87,0	9,1	3,9	100,0	
	Residuos	-0,2	0,2	0,1		
Sí	n	216	21	9	246	0,952*
	%	87,8	8,5	3,7	100,0	
	Residuos	0,2	-0,2	-0,1		
**Legumbres y frutas (ingesta diaria)**					n=323	
No	n	30	3	0	33	0,752*
	%	90,9	9,1	0,0	100,0	
	Residuos	0,6	0,1	-1,2		
Sí	n	253	25	12	290	0,752*
	%	87,2	8,6	4,1	100,0	
	Residuos	-0,6	-0,1	1,2		
**Refrescos o jugo artificial (frecuencia semanal)**					n=322*	
0	n	103	15	8	126	0,044
	%	81,7	11,9	6,3	100,0	
	Residuos	-2,5	1,6	2		
1 a 2	n	89	8	0	97	0,044
	%	91,8	8,2	0,0	100,0	
	Residuos	1,5	-0,2	-2,3		
3+	n	90	5	4	99	0,044
	%	90,9	5,1	4,0	100,0	
	Residuos	1,2	-1,5	0,2		

*Prueba Exacta de Fisher

El análisis de la relación entre las características relacionadas con el cáncer de mama y los subtipos moleculares de tumores presentados por las mujeres, expuesto en la Tabla 3, evidenció una diferencia estadísticamente significativa entre la edad al momento del diagnóstico y los subtipos moleculares de cáncer de mama (p=0,006). Las mujeres con edad inferior a 40 años presentaron predominantemente los subtipos luminal B y TN, mientras que aquellas con edades entre 40 y 59 años mostraron una mayor prevalencia de los subtipos luminal B y A.

Como se presenta en la Tabla 4, no se encontraron asociaciones estadísticamente significativas entre los subtipos moleculares de cáncer de mama y los hábitos de vida de las mujeres investigadas.

**Tabla 3 -:** Relación entre las características relacionadas con el cáncer de mama y los subtipos moleculares de cáncer de mama presentados por las mujeres participantes del estudio. São Paulo, SP, Brasil, 2023

		Subtipo molecular*				**Total**	**p-valor**
		**Luminal A**	**Luminal B**	**HER 2+**	**Triple negativo**		
**Edad al diagnóstico (a** ñ **os)**						n=317	
<40	n	13	29	16	20	78	0,006
	%	16,7	37,2	20,5	25,6	100,0	
	Residuos	-1,7	-1,3	0,9	2,8		
40 a 59	n	46	94	34	28	202	0,006
	%	22,8	46,5	16,8	13,9	100,0	
	Residuos	-0,5	1,4	-0,1	-1,2		
60+	n	16	15	4	2	37	0,006
	%	43,2	40,5	10,8	5,4	100,0	
	Residuos	3	-0,4	-1,1	-1,8		
**IMC** ^†^						n=317	
<25,0	n	27	48	14	17	106	0,389 ^‡^
	%	25,5	45,3	13,2	16,0	100,0	
	Residuos	0,5	0,4	-1,3	0,1		
25,0 a 29,9	n	32	49	27	16	124	0,389 ^‡^
	%	25,8	39,5	21,8	12,9	100,0	
	Residuos	0,7	-1,2	1,8	-1,1		
>=30	n	16	41	13	17	87	0,389 ^‡^
	%	18,4	47,1	14,9	19,5	100,0	
	Residuos	-1,4	0,8	-0,6	1,1		
**Familiares con cáncer de mama**						n=317	
0	n	41	84	35	36	196	0,048 ^‡^
	%	20,9	42,9	17,9	18,4	100,0	
	Residuos	-1,5	-0,3	0,5	1,6		
1	n	22	39	13	4	78	0,048 ^‡^
	%	28,2	50,0	16,7	5,1	100,0	
	Residuos	1,1	1,3	-0,1	-3		
2+	n	12	15	6	10	43	0,048 ^‡^
	%	27,9	34,9	14,0	23,3	100,0	
	Residuos	0,7	-1,2	-0,6	1,4		

*Expediente sin datos de la inmunohistoquímica de 6 mujeres; ^†^IMC = Índice de masa corporal; ^‡^Prueba Exacta de Fisher

Como se presenta en la Tabla 4, no se encontraron asociaciones estadísticamente significativas entre los subtipos moleculares de cáncer de mama y los hábitos de vida de las mujeres investigadas.

**Tabla 4 -:** Relación entre los hábitos de vida y los subtipos moleculares de cáncer de mama presentados por las mujeres participantes del estudio. São Paulo, SP, Brasil, 2023

		Subtipo molecular*				Total	p-valor
		Luminal A	Luminal B	HER 2+	Triple negativo		
**Consumo de bebida alcohólica**						n=317	
No	n	66	127	53	46	292	0,189 ^†^
	%	22,6	43,5	18,2	15,8	100,0	
	Residuos	-1,5	0	1,8	0		
Sí	n	9	11	1	4	25	0,189 ^†^
	%	36,0	44,0	4,0	16,0	100,0	
	Residuos	1,5	0	-1,8	0		
**Consumo de tabaco**						n=317	
Nunca	n	42	85	33	35	195	0,695 ^†^
	%	21,5	43,6	16,9	17,9	100,0	
	Residuos	-1,1	0	-0,1	1,3		
Exfumante	n	22	37	14	8	81	0,695 ^†^
	%	27,2	45,7	17,3	9,9	100,0	
	Residuos	0,9	0,5	0,1	-1,7		
Fumante	n	11	16	7	7	41	0,695 ^†^
	%	26,8	39	17,1	17,1	100,0	
	Residuos	0,5	-0,6	0	0,2		
**Tiempo de tabaquismo (a** ñ **os)**						n=122	
1 a 9	n	6	20	3	3	32	0,290 ^†^
	%	18,8	62,5	9,4	9,4	100,0	
	Residuos	-1,2	2,5	-1,4	-0,6		
10 a 19	n	7	11	4	4	26	0,290 ^†^
	%	26,9	42,3	15,4	15,4	100,0	
	Residuos	0	-0,1	-0,3	0,5		
20 o más	n	20	22	14	8	64	0,290 ^†^
	%	31,3	34,4	21,9	12,5	100,0	
	Residuos	1,1	-2,1	1,4	0,1		
**Actividad física (minutos por semana)**						n=317	
Insuficientemente activa (<150)	n	65	118	48	46	277	0,646 ^†^
	%	28,8	40,7	13,6	16,9	100,0	
	Residuos	0,5	-1	-0,2	1,1		
Suficientemente activa (>=150)	n	10	20	6	4	40	0,646 ^†^
	%	25	50	15	10	100,0	
	Residuos	-0,5	1	0,2	-1,1		
**Tipo de alimentación predominante a lo largo de la vida**							
**Alimentos industrializados (ingesta diaria)**						n=317	
No	n	13	23	8	8	52	0,992 ^†^
	%	25	44,2	15,4	15,4	100,0	
	Residuos	0,2	0,1	-0,3	-0,1		
Sí	n	62	115	46	42	265	0,992 ^†^
	%	23,4	43,4	17,4	15,8	100,0	
	Residuos	-0,2	-0,1	0,3	0,1		
**Az** úcar (ingesta di **aria)**						n=317	
No	n	22	33	6	9	70	0,072 ^†^
	%	31,4	47,1	8,6	12,9	100,0	
	Residuos	1,7	0,7	-2,1	-0,8		
Sí	n	53	105	48	41	247	0,072 ^†^
	%	21,5	42,5	19,4	16,6	100,0	
	Residuos	-1,7	-0,7	2,1	0,8		
**Leches y derivados de la leche (ingesta diaria)**						n=317	
No	n	18	35	10	14	77	0,690 ^†^
	%	23,4	45,5	13,0	18,2	100,0	
	Residuos	-0,1	0,4	-1,1	0,7	1	
Sí	n	57	103	44	36	240	0,690 ^†^
	%	23,8	42,9	18,3	15,0	100,0	
	Residuos	0,1	-0,4	1,1	-0,7		
**Legumbres y frutas (ingesta diaria)**						n=317	
No	n	6	13	7	7	33	0,607 ^†^
	%	18,2	39,4	21,2	21,2	100,0	
	Residuos	-0,8	-0,5	0,7	0,9		
Sí	n	69	125	47	43	284	0,607 ^†^
	%	24,3	44,0	16,5	15,1	100,0	
	Residuos	0,8	0,5	-0,7	-0,9		
**Refrescos o jugo artificial (frecuencia semanal)**						n=316 ^‡^	
0	n	36	53	16	19	124	0,451 ^†^
	%	29,0	42,7	12,9	15,3	100,0	
	Residuos	1,8	-0,3	-1,5	-0,2	93	
1 a 2	n	17	40	21	15	100,0	0,451 ^†^
	%	18,3	43,0	22,6	16,1		
	Residuos	-1,5	-0,2	1,8	0,1	99	
3+	n	22	45	16	16	100,0	0,451 ^†^
	%	22,2	45,5	16,2	16,2		
	Residuos	-0,4	0,4	-0,2	0,1		

*Expediente sin datos de la inmunohistoquímica de 6 mujeres; ^†^Prueba Exacta de Fisher; ^‡^Sin respuesta de 1 mujer

## Discusión

El análisis de los datos obtenidos en este estudio, que involucró a 323 mujeres, reveló asociaciones importantes entre características demográficas, hábitos alimentarios y los subtipos de cáncer de mama. Observamos que el no consumo de alimentos industrializados, así como el no consumo de refrescos o jugos artificiales, estuvo asociado a una menor proporción de carcinoma ductal. Además, las mujeres con menos de 40 años presentaron una mayor ocurrencia del subtipo de cáncer de mama triple negativo. Estos hallazgos refuerzan la influencia de la edad en el perfil molecular del cáncer de mama y destacan la relevancia de los factores dietéticos en los diferentes subtipos histológicos, subrayando la necesidad de integrar estas variables al desarrollar estrategias de prevención y políticas de salud eficaces para el manejo de la enfermedad.

Sin embargo, al interpretar estos resultados, es esencial considerar las limitaciones del estudio. La recolección de información, basada en autorreporte, presenta vulnerabilidades como imprecisiones en la medición y posibles sesgos de memoria, los cuales pueden variar según el rango de edad de las entrevistadas. Además, la representatividad de la muestra debe ser vista con cautela, ya que puede no reflejar completamente a la población general, dificultando la generalización de los resultados. El sesgo de supervivencia es otra preocupación relevante, dado que la inclusión exclusiva de mujeres diagnosticadas y tratadas puede distorsionar la relación observada entre hábitos de vida, características diagnósticas y la ocurrencia de subtipos de cáncer de mama. Esto puede llevar a la sobrestimación de las asociaciones, pues los casos con desenlaces desfavorables pueden estar subrepresentados.

La recolección de datos también se vio afectada por factores externos, como la pandemia causada por el virus SARS-CoV. La necesidad de adaptar los procedimientos de recolección, con la presentación de modificaciones a los comités de ética en investigación (CEP) de la *Escola de Enfermagem* y de la *Fundação Faculdade de Medicina* de la USP, generó retrasos y eliminó la posibilidad de realizar entrevistas presenciales. Esta modificación limitó el contacto directo entre investigador y participantes, potencialmente influyendo en la profundidad y la calidad de la información recolectada.

A pesar de estas limitaciones, los hallazgos sobre la distribución de los tipos de cáncer de mama son consistentes con datos poblacionales. La mayoría (87,6%) de los casos fue de carcinoma ductal, un resultado alineado con la prevalencia general de este tipo en la población^(13)^. Otros subtipos histológicos, como carcinoma mucinoso, carcinoma metaplásico, carcinoma medular y carcinoma micropapilífero, también fueron identificados, aunque son raros, cada uno representando menos del 5% de los casos, según se describe en la literatura^(4)^.

En cuanto a los subtipos moleculares, la predominancia de los tumores luminales en nuestro estudio refuerza la evidencia global de que corresponden a cerca del 80% de los casos de cáncer de mama, mientras que el subtipo triple negativo (TN) abarca aproximadamente el 10-15% de los diagnósticos^(14)^. Esta concordancia con datos internacionales fortalece la validez de nuestros resultados, indicando que el perfil molecular observado corrobora con la tendencia ya documentada en la literatura.

Además, el análisis de la variable “edad en el momento del diagnóstico” reveló que la mayoría de las participantes del estudio se encontraba en el rango de 40 a 59 años. De acuerdo con el INCA, la mayor concentración de exámenes de detección, como la mamografía, se realiza entre mujeres de 50 a 59 años, seguida del grupo etario de 60 a 69 años^(3)^. En contraste, la ACS recomienda iniciar el tamizaje a partir de los 40 años^(4)^. Estas diferencias en las recomendaciones subrayan la importancia de una discusión continua sobre la edad más adecuada para comenzar el tamizaje, teniendo en cuenta el contexto epidemiológico y las especificidades de la población estudiada.

Nuestro estudio identificó una asociación significativa entre la edad y los subtipos moleculares del cáncer de mama, con un aumento del riesgo de cáncer triple negativo (TN) en mujeres menores de 40 años. Esta observación es corroborada por un registro prospectivo en Brasil, que indicó una mayor proporción de tumores luminal B y TN en pacientes más jóvenes^(15)^. Además, la literatura ya establece que los cánceres TN se diagnostican de forma desproporcionada en mujeres más jóvenes^(16)^. Estos hallazgos destacan la importancia de incorporar la diversidad de los subtipos moleculares y la variabilidad en el riesgo entre diferentes rangos de edad al elaborar directrices de detección. Así, se vuelve esencial profundizar las investigaciones sobre cuál sería la edad más adecuada para iniciar el tamizaje, garantizando una detección temprana y eficaz en distintos grupos poblacionales.

Adicionalmente, el 39% de las mujeres de este estudio presentaron un índice de masa corporal (IMC) entre 25 y 29,9 kg/m², mientras que el informe de 2023 del Sistema de Vigilancia Alimentaria y Nutricional identificó que el 33,7% de la población adulta femenina brasileña tiene sobrepeso^(17)^. A pesar de ello, el análisis no reveló una asociación estadísticamente significativa entre el IMC y los subtipos histológicos o moleculares del cáncer de mama. Estos resultados están en consonancia con otro estudio que, aunque reconoce la relación entre un IMC elevado y el riesgo general de cáncer de mama, no encontró significancia al examinar esta relación con subtipos específicos de la enfermedad^(18)^. Cabe destacar que la literatura aún carece de estudios más detallados sobre estas asociaciones.

En lo que respecta a factores como el consumo de bebida alcohólica, el tabaquismo y la práctica de actividad física, no se identificaron asociaciones estadísticamente significativas en nuestro estudio, lo que difiere de lo ampliamente documentado. Por ejemplo, un análisis de una cohorte prospectiva estadounidense demostró una correlación significativa entre el consumo de alcohol y el aumento del riesgo de tumores con receptor hormonal positivo, sobre todo el carcinoma lobular, con efectos más pronunciados en mujeres posmenopáusicas^(19)^. Estos hallazgos respaldan la idea de la influencia de mecanismos hormonales en la relación entre el alcohol y el cáncer de mama, destacando el papel modulador del estrógeno^(19)^.

En cuanto al tabaquismo, un metaanálisis abarcador, que involucró datos de 14 estudios de cohorte, reveló un aumento general modesto (7%) en el riesgo de cáncer de mama entre las mujeres que fumaban en el momento del estudio^(20)^. Esta asociación fue particularmente relevante en cánceres que expresan el receptor de estrógeno (RE), con un riesgo incrementado del 76% para tumores RE positivos^(20)^. Además, el impacto del tabaquismo parece variar según la edad de inicio. Las mujeres que comenzaron a fumar antes del primer embarazo presentaron un riesgo aún mayor de desarrollar cáncer de mama^(20)^.

Estos resultados resaltan la complejidad de las interacciones entre los factores de estilo de vida y el riesgo de cáncer de mama, sugiriendo que la edad y el momento de la exposición a factores como el tabaco desempeñan roles fundamentales en la carcinogénesis mamaria. Por lo tanto, son necesarias investigaciones futuras para comprender mejor estas dinámicas e informar estrategias preventivas personalizadas.

Contrario a los hallazgos del presente estudio, que no encontró significación estadística en la relación entre la práctica de actividad física y los subtipos histológicos o moleculares del cáncer de mama, un estudio caso-control realizado en Alemania demostró una asociación inversa significativa entre la práctica de actividad física y el riesgo de cáncer de mama en la fase posmenopáusica^(21)^. Los autores informaron que el efecto protector fue más pronunciado en carcinomas invasivos con receptor hormonal positivo^(21)^. Además, se verificó que la actividad física realizada después de los 50 años resultó ser más eficaz en la prevención de carcinomas RE+ y RP+ en mujeres posmenopáusicas que la práctica en edades más jóvenes^(21)^.

En el ámbito del presente estudio, se logró establecer significación estadística en las relaciones entre los subtipos histológicos y algunas variables relacionadas con los hábitos alimentarios, específicamente la ingesta de alimentos industrializados (p=0,003) y el consumo de refrescos o jugos artificiales (p=0,044) (Tabla 2). Con respecto a la dieta, la literatura científica presenta resultados heterogéneos. Algunos estudios sugieren que la enzima aromatasa, presente en el tejido adiposo, puede convertir el colesterol circulante en estradiol, aumentando los niveles de esta hormona en los tejidos mamarios e influyendo en la expresión de los receptores de estrógeno, lo que puede afectar el comportamiento de las células cancerígenas^(22)^. Además, investigaciones revelan que dietas ricas en alimentos curados pueden estar asociadas a un riesgo aumentado de cáncer de mama receptor hormonal negativo, mientras que el consumo de alimentos asados se correlaciona con un mayor riesgo de cáncer de mama receptor hormonal positivo^(23)^.

El papel de los alimentos industrializados también merece destaque. Estos alimentos, incluyendo los procesados y embutidos, contribuyen a una dieta proinflamatoria, mientras que una alimentación rica en frutas, vegetales y granos integrales se asocia a menores niveles de biomarcadores inflamatorios^(24)^. Por otro lado, el consumo elevado de carne roja y procesada se relaciona con un aumento de dichos marcadores inflamatorios^(24)^. En este estudio, se observaron diferencias significativas en el consumo de alimentos industrializados asociadas al carcinoma ductal. Específicamente, la frecuencia de carcinoma ductal fue menor entre mujeres que reportaron abstenerse del consumo de alimentos industrializados y de refrescos o jugos artificiales, con valores de residuos de -2,6 y -2,5, respectivamente (p=0,044). Esto sugiere que la ausencia de consumo de dichos productos puede estar asociada a una menor incidencia de carcinoma ductal.

Sin embargo, no se encontró significación estadística al analizar las asociaciones entre el consumo de alimentos industrializados, refrescos o jugos artificiales y los subtipos moleculares. Aunque otros estudios han reportado una relación entre dietas proinflamatorias y subtipos de cáncer de mama positivos para receptor de estrógeno o de progesterona, así como para el receptor HER2, esta asociación no se observó en el caso del subtipo triple negativo (TN)^(24)^.

Es importante señalar que la literatura aún es escasa en lo que respecta a la caracterización de los subtipos histológicos del cáncer de mama, dedicándose mayor énfasis a los subtipos moleculares. Aunque este estudio es de naturaleza transversal, lo que impide la inferencia de causalidad, nuestros hallazgos proporcionan evidencias significativas que contribuyen a la generación de hipótesis y a la identificación de factores de riesgo o marcadores que merecen investigaciones más profundas. Estudios transversales, como este, son fundamentales para plantear hipótesis y sugerir direcciones para futuras investigaciones, especialmente mediante estudios longitudinales o experimentales, que podrían esclarecer las relaciones causales.

En resumen, aunque las asociaciones observadas no establecen una relación directa de causa y efecto, los resultados sugieren conexiones relevantes entre los hábitos de vida, las características diagnósticas y los subtipos de cáncer de mama. Estos hallazgos tienen el potencial de orientar futuras investigaciones y contribuir significativamente al avance del conocimiento en el área. Además, nuestros resultados ofrecen un valioso soporte para la práctica de la Enfermería, subrayando la importancia de promover estilos de vida saludables y brindar asistencia calificada a las mujeres en tratamiento o seguimiento por cáncer de mama. Se destaca la necesidad de estudios adicionales, especialmente en la población brasileña, para desarrollar estrategias preventivas eficaces contra la enfermedad.

## Conclusión

Este estudio buscó investigar la compleja relación entre las características de las mujeres relacionadas con el cáncer de mama, sus hábitos de vida y la incidencia de diferentes subtipos de la enfermedad. Los resultados obtenidos contribuyen de manera significativa a la comprensión de estas interacciones y su influencia en la manifestación de los subtipos de cáncer de mama.

Se verificó una asociación significativa entre la edad de las mujeres en el momento del diagnóstico y los subtipos moleculares del cáncer de mama, determinados por inmunohistoquímica. Específicamente, las mujeres con menos de 40 años presentaron una mayor frecuencia de desarrollar cáncer de mama triple negativo (TN).

Nuestros hallazgos destacan asociaciones entre hábitos de vida, como el consumo de alimentos industrializados y de refrescos o jugos artificiales, y los subtipos histológicos del cáncer de mama. Esto resalta la importancia de considerar dichos factores en la prevención de la enfermedad.

El presente estudio contribuyó de manera sustancial a los campos de la enseñanza, la investigación y la asistencia en Enfermería, al conferir mayor visibilidad a los hábitos de vida modificables identificados como factores de riesgo para el cáncer de mama. Proporciona información para orientar estrategias de salud pública en la prevención, el diagnóstico precoz y la implementación de políticas eficaces para mitigar el impacto del cáncer de mama en la sociedad.
